# A brain cyst load-associated antigen is a *Toxoplasma gondii* biomarker for serodetection of persistent parasites and chronic infection

**DOI:** 10.1186/s12915-021-00959-9

**Published:** 2021-02-09

**Authors:** Céline Dard, Christopher Swale, Marie-Pierre Brenier-Pinchart, Dayana C. Farhat, Valeria Bellini, Marie Gladys Robert, Dominique Cannella, Hervé Pelloux, Isabelle Tardieux, Mohamed-Ali Hakimi

**Affiliations:** 1 Team Host-Pathogen Interactions and Immunity to Infection, Institute for Advanced Biosciences, INSERM U1209, CNRS UMR5309, Université Grenoble Alpes, Grenoble, France; 2 Laboratory of Parasitology and Mycology, Grenoble Alpes University Hospital, CS10217, 38043 Grenoble, France; 3 Team Membrane and Cell Dynamics of Host Parasite Interactions, Institute for Advanced Biosciences, INSERM U1209, CNRS UMR5309, Université Grenoble Alpes, Grenoble, France

**Keywords:** *Toxoplasma gondii*, Toxoplasmosis, Biomarker, Chronic infection, Serology, Recombinant antigen

## Abstract

**Background:**

Biomarker discovery remains a major challenge for predictive medicine, in particular, in the context of chronic diseases. This is true for the widespread protozoan *Toxoplasma gondii* which establishes long-lasting parasitism in metazoans, humans included. This microbe successively unfolds distinct genetic programs that direct the transition from high to low replicative potential inside host cells. As a slow-replicating cell, the *T. gondii* bradyzoite developmental stage persists enclosed in a cyst compartment within tissues including the nervous system, being held by a sustained immune equilibrium which accounts for the prolonged clinically silent phase of parasitism. Serological surveys indicate that nearly one third of the human population has been exposed to *T. gondii* and possibly host bradyzoites. Because any disruption of the immune balance drives the reverse transition from bradyzoite to fast replicating tachyzoite and uncontrolled growth of the latter, these people are at risk for life-threatening disease. While serological tests for discriminating recent from past infection are available, there is yet no immunogenic biomarker used in the serological test to allow ascertaining the presence of persistent bradyzoites.

**Results:**

Capitalizing on genetically engineered parasites induced to produce mature bradyzoites in vitro, we have identified the BCLA/MAG2 protein being restricted to the bradyzoite and the cyst envelope. Using laboratory mice as relevant *T. gondii* host models, we demonstrated that BCLA/MAG2 drives the generation of antibodies that recognize bradyzoite and the enveloping cyst structure. We have designed an ELISA assay based on a bacterially produced BCLA recombinant polypeptide, which was validated using a large collection of sera from mice of different genetic backgrounds and infected with bcla+ or bcla-null cystogenic and non-cystogenic *T. gondii* strains. To refine the design of the ELISA assay, we applied high-resolution BCLA epitope mapping and identified a specific combination of peptides and accordingly set up a selective and sensitive ELISA assay which allowed the detection of anti-BCLA/MAG2 antibodies in the sera of human patients with various forms of toxoplasmosis.

**Conclusions:**

We brought proof of principle that anti-BCLA/MAG2 antibodies serve as specific and sensitive serological markers in the perspective of a combinatorial strategy for detection of persistent *T. gondii* parasitism.

**Supplementary Information:**

The online version contains supplementary material available at 10.1186/s12915-021-00959-9.

## Background

Chronic diseases represent a major and ever-increasing burden to public health worldwide, and this by causing the expansion of social costs involving long-term treatments and management, and by worsening the disability-adjusted life years (DALYs) global health metrics. In this equation, the contribution of phylogenetically unrelated infectious agents including viruses, bacteria, helminths, and protozoan parasites has already been ascertained. The protozoan *T. gondii* is one of the most widespread microbes on Earth endowed with the striking ability to establish long-lasting parasitism in virtually all warm-blooded metazoans [1]. Humans are no exception, as nearly one third of the worldwide population is reported to be *T. gondii* sero-positive and presumed infected. The infection starts with the ingestion of host-to-host transmissible zoite stages, which once they reach the intestinal epithelium, transform into replicative tachyzoites. While the acute expansion of the tachyzoite population is usually restrained by the host immune system [2], the latter also directs the differentiation of few tachyzoites into slow-replicating and host-to-host transmissible bradyzoites. Indeed, these bradyzoites reside within intracellular compartments called cysts within tissues including the brain, the retina, and the skeletal muscles, being held *quasi* silent by a IFNγ-orchestrated immune response [3]; hence, they account for the subsequent prolonged and so-called clinically silent phase of parasitism. Disruption of this immune balance drives the reverse transition from bradyzoite to tachyzoite and uncontrolled growth of the latter, thereby causing life-debilitating and threatening damages in the aforementioned tissues, and exemplified with the encephalitis of AID patients [4]. Accordingly, recurrent severe ocular diseases are common pathologies in particular for congenitally acquired *T. gondii* infections [5], while an increased risk of schizophrenia-related mental disorders has been also proposed to stem from bradyzoite persistence in the brain [6]. In addition, a significant risk of subsequent systemic or/and chronic-related illnesses exists for the ever-increasing number of patients undergoing transient or sustained immunosuppressive therapies [7].

Therefore, the management of this large set of clinical situations would certainly benefit from a non-invasive test that is able to recognize the reactivation of tissue cysts or optimally the extent of the reactivation. Furthermore, given the high prevalence of *T. gondii* in low-income and developed countries [8], there is an identified need to set up cost-effective, sensitive, and specific assays for detecting *T. gondii* bradyzoite/cyst across all age classes of the human population. In principle, serology-based assays offer the potential of low-cost, accurate, and rapid testing of microbe exposure, and these are routinely used worldwide for the control of recent or past *T. gondii* infection in pregnant woman, unborn baby, or immunocompromised patients. Because the large majority of IgGs from sero-positive humans recognize the tachyzoite proliferative stage with minor reactivity reported for the cyst and bradyzoite-like stage [9], there is a presumption for chronic infection but direct serological evidence pertaining to the cystic stage are still missing.

This lack of knowledge stems in large part from the limited information on the bradyzoite biology, a cryptic developmental stage that unlike the proliferating tachyzoite, cannot be continuously propagated in vitro. With regard to biological features of the cyst-loaded bradyzoites, pioneering work has uncovered the development of secondary cyst foci following the fission of the primary one or through the escape of bradyzoites from the mother cyst and further re-establishment in other cells [10, 11]. Recent evidence of periodic and transient growth within tissue cysts in vivo [12] probably through asynchronous endodyogeny and endopolygeny multiplication processes [10] also supports the view of an active bradyzoite phase of parasitism, which thus would imply dynamic co-adjustments between parasite and host immune functions. While contributing to maintain parasite loads in tissues, the uncovered dynamic status of bradyzoite must be now integrated into the risk estimation of pathologies related to *T. gondii* chronic infection.

As regards the biomarkers for the bradyzoite developmental stage, many attempts over the last decades to design ex vivo protocols to induce tachyzoite to differentiate into bradyzoite have set the first basis for identifying specific protein markers for both stages. Aside from these ex vivo-derived parasites, the laboratory mouse has remained the most reliable source of cyst loaded with mature bradyzoites, since mice are well-acknowledged as among the most relevant wild-living hosts for *T. gondii* [13].

Collectively, studies using these biological samples have highlighted the drastic change of the parasite’s gene expression profiles along with the tachyzoite to bradyzoite developmental transition, a gene pattern that reveals major changes in the parasite metabolism, but also the remodeling of the parasite surface with the restricted expression of stage-specific surface proteins, and the assembly of the cyst wall [3]. Among the hallmarks for bradyzoite proteins or cyst wall, critical components are respectively the BAG1 protein [14] and the CST protein family [15–18], but despite their immunogenicity, none of these interesting biomarkers has been assessed for serological diagnosis yet.

The increasing interest in deciphering the molecular base of bradygenesis has highlighted not only the host cell physiological status as an important determinant [19] but also underlined the key contribution of chromatin shapers, including histone modifications, in directing epigenetic programs specifying given developmental stage [20–23]. Recently, the *T. gondii* microrchidia (MORC) protein was identified as a transcriptional repressor that regulates parasite developmental trajectories. This finding allowed developing a new and robust strategy to analyze in vitro bradyzoite- and cyst-resident proteins based on genetically engineered cystogenic parasites for which the MORC can be acutely and conditionally depleted [24].

In a search for additional parasite immunogenic targets that would be specific of cyst-containing bradyzoites, we capitalized on the MORC expression-based strategy for stage discrimination. We identified a specific cyst-resident protein, hereafter referred as BCLA (Brain Cyst Load-associated Antigen), independently reported as the matrix antigen 2 (MAG2) [17, 18], the expression of which is regulated at the transcriptional level by MORC. This “BCLA/MAG2” drives a robust humoral response post-acute infection and over the chronic infection phase in mice, which can be ascertained by a long-lasting IgG production. These BCLA/MAG2 antigenic features were further detailed as well as validated for humans. This study has led to the first proof of concept stating that “BCLA/MAG2” would be a valuable component in the perspective of a toolkit designed to assist the diagnosis of *T. gondii* cysts in host organs for achieving better disease management in veterinary and human medicine.

## Results

### Depletion of MORC phenocopies the inhibition of HDAC3 in inducing the expression of bradyzoite- and cyst-associated immunodominant antigens

**Fig. 1:**
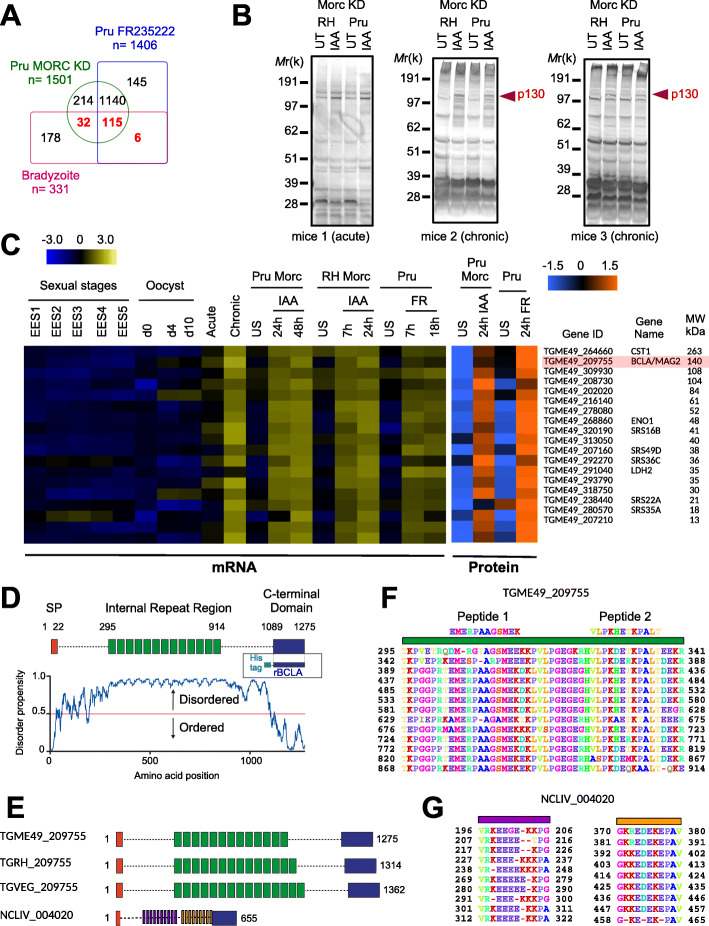
Depletion of MORC phenocopies the inhibition of HDAC3 by inducing the expression of bradyzoite stage-specific genes. **a** Venn diagram illustrating the overlap between the MORC-regulated genes in RH and in Pru strains, and the 331 genes whose expression were reported to be exclusive to bradyzoite. **b** Expression of protein upon MORC depletion in both type I (RH*ku80*) and type II (Pru*ku80*) intracellular tachyzoites. Samples were taken 24 h after the addition of IAA and were probed with the sera from mice during acute (5 days) or chronic (> 5 weeks) toxoplasmosis. The experiment was repeated three times, and representative blots are shown. **c** Heat map showing the clustering analysis of selected bradyzoite genes and their corresponding proteins that are differentially regulated after the depletion of MORC for different combinations of strain and induction. Parasites were left unstimulated (US) or treated with FR235222 (FR) to inhibit HDAC3. The abundance of their transcripts in the various stages of development, namely early and mature bradyzoites, enteroepithelial stages (EESs), or three developmental stages of oocysts, is shown. The color scale indicates log2-transformed fold changes. **d** Chart illustration showing the disorder score as a function of protein amino acid position (generated via the IUPred server). The C-terminal domain end of BCLA (residues 1089 to 1275, hereafter referred to as rBCLA) is predicted as structured as opposed to the rest of the protein-containing core repeated motifs (in green). **e** Chart illustration showing the structural motifs of *Toxoplasma gondii* (TG) and *Neospora caninum* (NC) BCLA proteins. **f** Alignment of the 13 central repeats of the BCLA protein of the ME49 strain. Peptides 1 and 2 have served to direct antibodies against BCLA in rabbits. **g** Alignment of repeats of the BCLA protein of *Neospora caninum* which is characterized by two distinct families each comprising 10 repeated elements

We have already reported that the *T. gondii* in vitro conversion of the intracellular dividing tachyzoites into bradyzoites could be achieved through the pharmacological inactivation of HDAC3 using the HDAC inhibitor FR235222 [20–22]. We also recently documented that HDAC3 teams up with a microrchidia (MORC) homolog which promotes the recruitment of HDAC3 to the chromatin [24]. Accordingly, the conditional knockdown of MORC in *T. gondii* tachyzoites engineered to endogenously express the chimeric MORC-HA-Auxin-inducible degron phenocopied HDAC3’s inhibition by inducing about half of the genes known to be expressed exclusively in the bradyzoite stage (Fig. 1a) [24]. Therefore, we assumed that switching-off MORC would provide a good base to test whether anti-*T. gondii*-positive sera contain antibodies other than those directed against tachyzoite antigens and traditionally used in serological tests that could specifically target bradyzoite and cyst antigens. Using SDS PAGE and Western blot, we analyzed the reactivity profile of the sera from BALB/c mice collected post-inoculation of the wild-type and notoriously high cyst-producing type II strain (76K) during the acute or the chronic phases of toxoplasmosis. Upon MORC depletion only, the sera from mice undergoing chronic infection allowed identifying a protein of an apparent approximate mass ~ 130 kDa (Fig. 1b). Relying on the mass spectrometry-based proteomic datasets that we had generated to compare the effects of MORC depletion with those of HDAC3 inhibition on the parasite proteome [24], we selected protein candidates of the appropriate mass based on their restricted bradyzoite-specific expression profile. Ranking of the top differentially expressed and the most abundant proteins with respect to their molecular weight allowed highlighting a single protein encoded by *TGME49_209755* with a predicted size of 140 kDa, which we named brain cyst-loaded antigen (BCLA) (Fig. 1c). Importantly, the transcriptomics data analysis matched well with those derived from the proteomic analysis with the increase of BCLA protein amounts upon MORC depletion or HDAC3 inhibition “quantified” by the proteomic analysis (Fig. 1c) and was validated by the detection of both the cyst wall protein CST1 and the bradyzoite hallmark metabolic enzymes LDH2 and ENO1 (Fig. 1c).

### The expression of *bcla* is induced by the degradation of MORC, as well as through the FR235222-induced inhibition of HDAC3

The BCLA protein in the type II ME49 strain has a predicted N-terminal signal peptide and a conserved C-terminal region of ~ 150 residues that border a central core domain typified by a motif of 48 amino acids repeated 13 times (Fig. 1d and Additional file 1: Figure S1). While these repeats differ in their number among the *T. gondii* canonical lineages, they also show divergence and shorter length in the BCLA homologous protein of the closely related Coccidian *Neospora caninum* (Fig. 1e–g). BCLA is quite conserved in *Hammondia hammondi* but partial DNA sequencing does not allow to predict the number of internal repeats within the protein (Additional file 1: Figure S1). One of the main predictive structural features for BCLA was its disorder propensity detected throughout its sequence including the core repeated motifs (Fig. 1d) except at the C-terminal end (approximately from aa 1089 to 1275) which is predicted as structured and may constitute a separate domain (Fig. 1d).

**Fig. 2:**
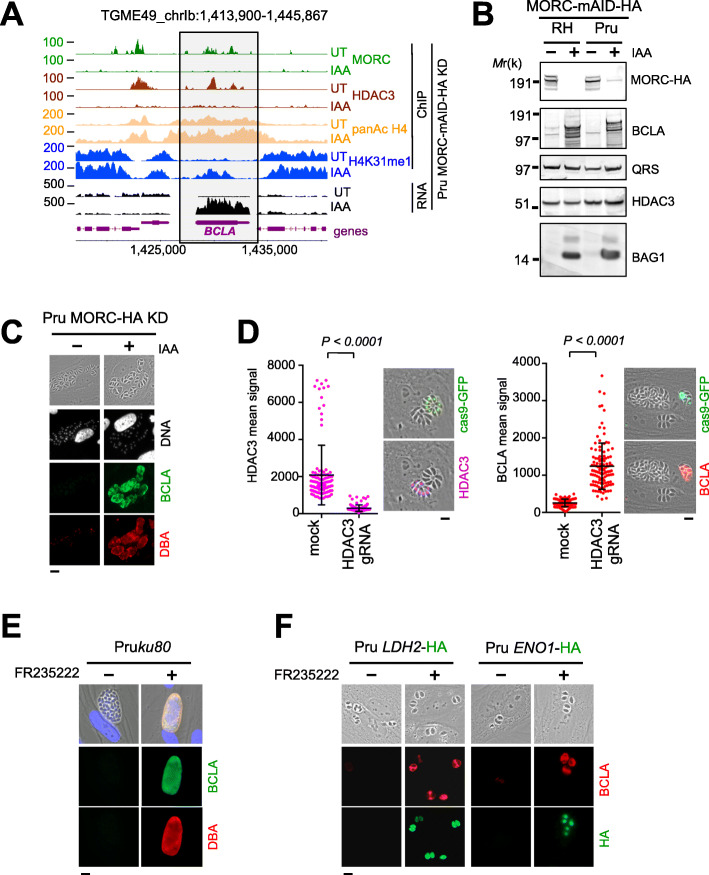
Degradation of MORC, similar to the inhibition of HDAC3, induces the expression of *Bcla.* **a** Density profiles are displayed over representative regions of Chr. Ib. Chromosomal positions are indicated on the *x*-axis. The ChIP-seq profiles were obtained using antibodies directed against various histone marks, HDAC3 and HA (MORC detection), and chromatin sampled from a Pru MORC KD strain that was either untreated (UT) or treated with IAA for 24 h. Read density from ChIP-seq data and RPKM values for RNA-seq data (in black) are shown on the *y*-axis. The *Bcla* gene is shown in purple. **b** Expression of BCLA protein upon MORC depletion in both type I (RH*ku80*) and type II (Pru*ku80*) intracellular tachyzoites. Samples were taken 24 h after addition the of IAA and were probed with antibodies against HA (MORC detection), BCLA (rabbit sera against BCLA peptides 1 and 2), BAG1, and HDAC3. QRS was used to control parasite loading. The experiment was repeated three times, and a representative blot is shown. **c** Expression levels of cyst-specific markers after depletion of MORC in the Pru strain were measured by IFA. Fixed and permeabilized parasites were probed with antibodies against BCLA (green) along with DBA lectin (red). Scale bar, 10 μm. **d** CRISPR-mediated genetic inactivation of *hdac3* promotes a severe reduction of HDAC3 (purple) and a concomitant induction of BCLA (red). The efficiency of genetic disruption in Cas9-expressing parasites was monitored by cas9-GFP expression (in green). Graph on the left: in situ quantification of nuclear HDAC3 or vacuolar BCLA using IFA. The horizontal bars represent the mean ± s.d. of the nuclear MORC intensity from three independent experiments. The *P* values were calculated using one-way ANOVA. Scale bar, 5 μm. **e** Expression of BCLA was monitored following HDAC3 chemical inactivation by FR235222 and BCLA (green, rabbit sera against BCLA peptides 1 and 2)/DBA (red) co-staining. Scale bar, 10 μm. **f** Pru strains were engineered to endogenously epitope tag with HA two bradyzoite-specific markers, *LDH2* and *ENO1*. Expression was monitored following HDAC3 chemical inactivation by FR235222 and BCLA (red, rabbit sera against BCLA peptides 1 and 2)/HA (green) co-staining. Scale bars, 10 μm. Experiments were conducted five times, and representative images are displayed

At the genomic level, in the MORC-depleted cell context, a substantial reduction in the chromatin occupancies of both HDAC3 and MORC was observed nearby the *TGME49_209755/Bcla* gene (Fig. 2a). In this regard, the loss of HDAC3’s binding resulted in increased levels of histone acetylation at the promoter region that caused the transcriptional activation of *Bcla* (Fig. 1c and Fig. 2a). This acetylation pattern was indeed a characteristic of the genes directly regulated by MORC [24]. To further study the dynamics and the subcellular distribution of BCLA during infection, we raised rabbit antisera against synthetic non-overlapping peptides located at the extreme end of the conserved repeat of BCLA (Fig. 1f). Exposing intracellular dividing *T. gondii* tachyzoites to indole-3-acetic acid (IAA) prior to analyzing protein expression profile allowed discriminating a protein compatible with the BLCA molecular weight at ~ 140-kDa in the IAA-treated sample, an observation consistent with the neosynthesis of BCLA (Fig. 2b). Of note, anti-BCLA antibodies recognized multiple lower molecular weight polypeptides, which could account for multiple BCLA protein processing (Fig. 2b), a pattern also seen when cell extracts were probed with sera collected during the chronic phase of infection (middle blot of Fig. 1b).

Along with BCLA, MORC-depleted parasites concomitantly expressed the bradyzoite (BAG1; Fig. 2b) canonical marker and showed clear reactivity with *Dolichos biflorus lectin* (DBA), a specific feature of the cyst wall (Fig. 2c). Consistently, CRISPR-mediated *hdac3* gene disruption (Fig. 2d) and HDAC3’s chemical inhibition (Fig. 2e, f) both induced unequivocal co-synthesis of BCLA with the LDH2 and ENO1 bradyzoite hallmark metabolic enzymes (Fig. 2f). Furthermore, RNA-seq analysis strengthened the view of BCLA’s synthesis being restricted to the bona fide bradyzoite stage in vivo; indeed, *bcla* transcripts were largely detected in the brain samples collected during chronic toxoplasmosis while merely being detected in tachyzoites during the acute phase and were undetectable in stages that specifically develop in feline intestines prior to sexual commitment (Fig. 1c).

### The neosynthesized BCLA is secreted into the PV and delineates the PVM

**Fig. 3:**
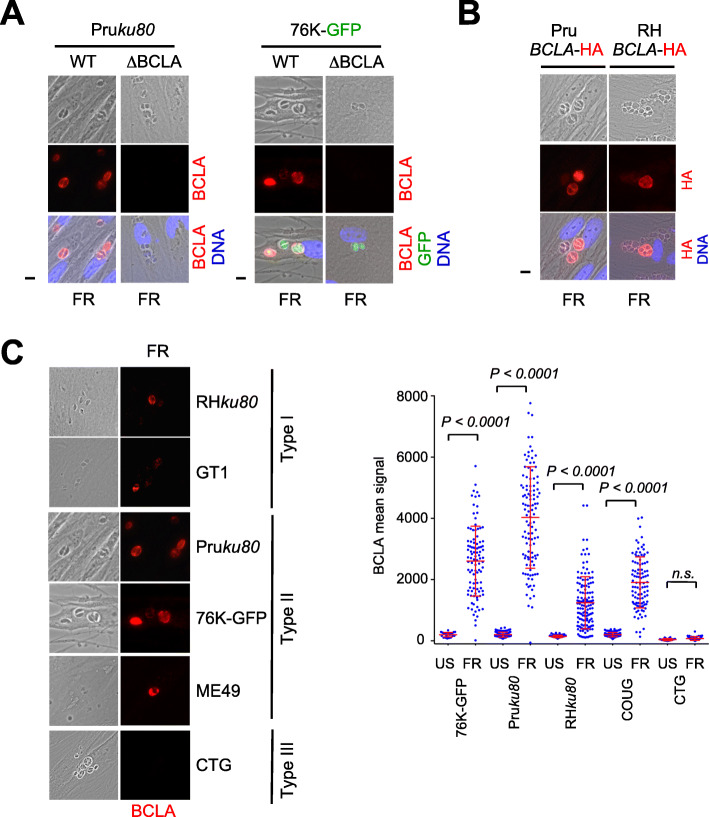
FR235222-mediated inhibition of HDAC3 induces BCLA in a strain-specific manner. **a** HFFs were infected with WT or Δ*bcla* parasites (Pruku80 or 76K), and expression of BCLA was monitored following HDAC3 chemical inactivation by FR235222 and BCLA (red) staining. Cells were co-stained with Hoechst DNA-specific dye (blue) or GFP (green). BCLA is not labeled in Δ*bcla* strains, therefore validating the specificity of the anti-BCLA antibodies. Scale bar, 10 μm. **b** Pru*ku80* or RH*ku80* strains were engineered to endogenously epitope tag with HA BCLA. Expression was monitored following HDAC3 chemical inactivation by FR235222 following by HA and Hoechst DNA-specific dye co-staining. The experiment was conducted more than three times, and representative images are displayed. Scale bars, 10 μm. **c** Expression of BCLA (red) following HDAC3 chemical inactivation by FR235222 was measured by IFA in HFFs infected with type I (RHΔ*ku80* and GT1), type II (PruΔ*ku80*, 76K-GFP-luc, and ME49), and type III (CTG) strains of *T. gondii*. Scale bar, 10 μm. Right graph: quantification of the intensity of BCLA in each PV after FR235222 stimulation. Each symbol marks the BCLA density of a single PV. The results are represented as mean ± standard deviations from two independent experiments; the number of PVs quantified was at least 70. Statistical significance when comparing each individual FR235222-treated strain and the corresponding control (DMSO, 0.1%) was determined by a non-parametric Mann-Whitney test

Upon MORC-depletion or HDAC3-inhibition, BCLA was readily detected in the vacuolar space and accumulated overtime at the PV membrane (PVM) vicinity, as evidenced by the co-staining with DBA (Fig. 2e). Conversely, BCLA was no longer detected in cells infected with tachyzoites genetically engineered to lack *bcla* (Δ*bcla*, Additional file 2: Table S1), thereby validating the in-house antibody specificity (Fig. 3a). Finally, when we monitored BCLA distribution in type I (RHΔ*ku80*) and II (PruΔ*ku80*) lines expressing the endogenous protein in fusion with the HA-Flag tags and using anti-HA antibodies in situ, we showed that once stimulated by the HDAC3 inhibitor, FR235222, the HA-tagged BCLA protein was targeted to the vacuolar space and in the vicinity of the vacuole membrane regardless of the strain type (Fig. 3b). Thus, the presence of the C-terminal fusion tag did not affect the subcellular localization of BCLA, which was found to be similar to the one in the untagged strain when assessed with the anti-BCLA sera (Fig. 3a, b). While exposing different parasite strains of *T. gondii* to FR235222, we uncovered that the intensity of the BCLA expression signal greatly varies depending on the infecting strains, ranging from a very strong induction in type II (Pru, ME49, 76K) strains to a rather moderate one with type I (GT1 and RH) and haplogroup 11 (COUG) strains, to a surprisingly non-detectable level in cells infected by the type III (CTG) strain (Fig. 3c). The lack of signal in CTG is probably not a type III lineage-specific trait but rather a peculiarity of this strain, which remains to be explored. Peptidic sequence divergence of the antigen cannot explain this disparate phenotype as they are found conserved over all strain types (Additional file 2: Table S1). The level of the FR235222-induced BCLA likely mirrors the cystogenic potential of the parasite strains, hence culminating in the case of type II strains.

### *Bcla*-deficient parasites maintain the ability to develop chronic infection in mice

**Fig. 4:**
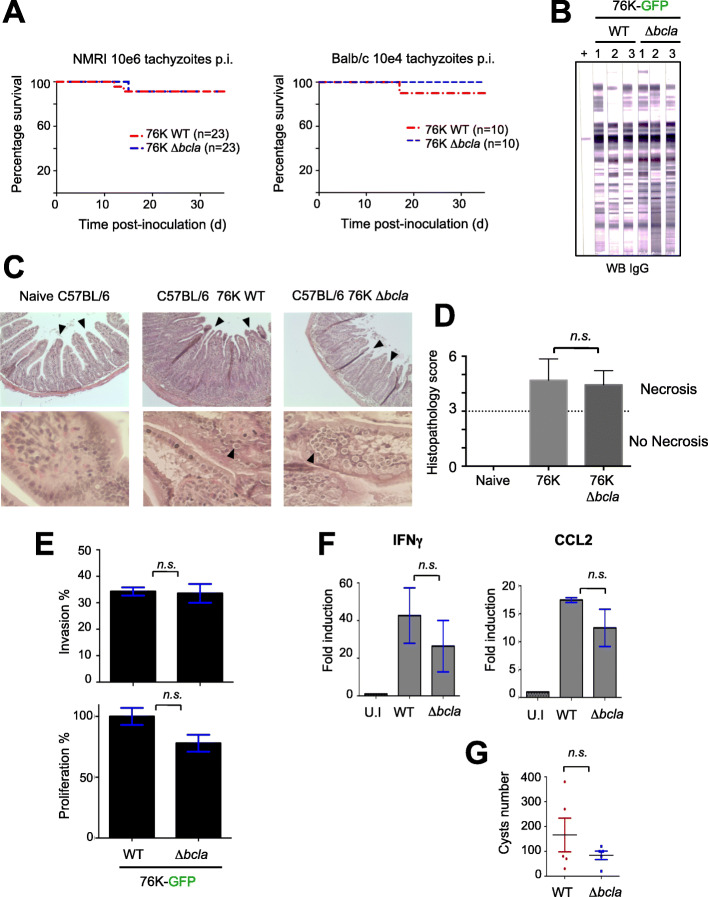
BCLA deficiency does not significantly impact the infectious potential. **a** NMRI (*n* = 23) or Balb/c (*n* = 10) mice were given 10^6^ and 10^4^ tachyzoites, respectively, of the 76K-GFP-luc WT and Δ*bcla* strains intraperitoneally and their survival was monitored. At day 5 post-inoculation, all the mice displayed clinical signs (weight loss and ruffled fur). Significance was tested using log-rank Mantel–Cox (NMRI: *P* = 0.9546; Balb/c: *P* = 0.3173) and Gehan–Breslow–Wilcoxon (NMRI: *P* = 0.9283; Balb/c: *P* = 0.3173) tests. **b** All the NMRI mice that survived the i.p. inoculation with Δ*bcla* and the WT strain showed sero-conversion when serologically tested by Western blot (IgG, LD bio Diagnostic), 10 weeks post-infection. **c** Histological analysis of C57BL/6 mouse ilea 8 days post-cyst ingestion. The ilea of non-infected mice (*n* = 4) are characterized by a well-defined epithelial architecture and villous morphology. Mice infected with either WT (*n* = 4) or *bcla*-deficient (*n* = 4) cysts are typified by a severe necrosis of the villi and mucosa compared to the non-infected mice. Bar, 100 μm. These experiments were repeated twice with similar results. **d** Histopathological scores in the ilea of mice (*n* = 4) infected with WT cysts were significantly lower compared with bcla-deficient-infected mice (*n* = 4). The horizontal line indicates the border between mild inflammation (< 3) and necrosis (> 3). Data are given as means ± SD, and *P* values were determined by the Mann-Whitney test. **e** Parasites lacking *bcla* exhibit no growth defect in vitro as determined by fluorescence imaging assays in HFFs. Vacuoles and parasites were counted to determine both the invasion and the proliferation indexes. Data are mean value ± s.d. of triplicates from two independent experiments. *P* values were calculated using a non-parametric Mann-Whitney test. n.s, not significant. **f** Quantitative RT-PCR analysis of IFNγ and CCL2 mRNA in ilea of C56BL/6 mice orally infected 8 days earlier. The results are represented as mean ± standard deviations from at least two independent experiments. *P* values were calculated using a non-parametric Mann-Whitney test. n.s, not significant. **g** Cysts harvested from the brains of NMRI mice orally infected 8–10 weeks earlier were enumerated using microscopy. The results are represented as mean ± standard deviations from at least two independent experiments. *P* values were calculated using a non-parametric Mann-Whitney test. n.s, not significant

To get functional insights on the BCLA protein, we next inoculated NMRI and BALB/c mice with different doses of tachyzoites from the cystogenic 76K strain either encoding or deleted for the single open reading frame of the *bcla* gene (Additional file 2: Table S1). For both WT and Δ*bcla* inocula, mice showed similar clinical signs such as weight loss and ruffled fur but eventually survived the infection (Fig. 4a). Accordingly, these mice mounted proper adaptive immune responses (Fig. 4b), irrespective of the route of inoculation, the mouse genotype, and the inoculum size (Fig. 4a).

### *Bcla* deficiency affects the integrity of brain cysts isolated from chronically infected mice

**Fig. 5:**
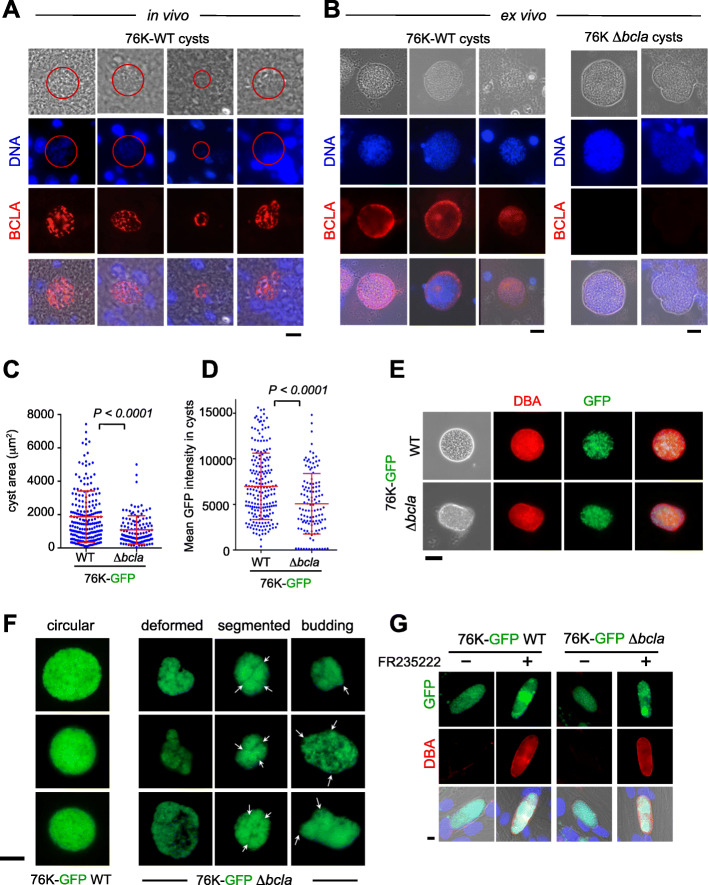
BCLA localizes within the cyst matrix and at the cyst periphery while *bcla* disruption induces subtle changes in cyst morphology. **a**, **b** BCLA (red) and DNA (blue) were detected in histological sections of brains (**a**, in vivo) or in cysts purified from brains (**b**, ex vivo) in mice chronically infected with 76K-GFP-luc WT or Δ*bcla* strains. Scale bar, 10 μm. **c**, **d** Comparative analysis of the area (**c**) and the GFP-fluorescence intensity (**d**) of WT and Δ*bcla*-containing cysts purified from the brains of NMRI mice that survive to challenge (in Fig. 4a) showed that loss of *bcla* translated into smaller cysts with reduced parasite load. **e** DBA staining (red) of the glycosylated wall from cysts isolated from mice chronically infected with 76K-GFP-luc WT or Δ*bcla* strains. Scale bar, 40 μm. **f** Representative panels of Δ*bcla*-containing cysts harboring deformations, segmentations, and “buds” compared to the round and well-defined WT cysts. Scale bar, 40 μm. **g** DBA (red) staining of the glycosylated membrane surrounding in vitro-converted bradyzoites. HFFs were infected with 76K-GFP-luc or 76K-GFP-luc-Δ*bcla* tachyzoites and treated with vehicle (DMSO) or low dose of FR235222 (25 ng/mL for 7 days). Lectin DBA labeling is similar in the membranes surrounding in vitro-converted bradyzoites of Δ*bcla* parasites and the WT strain. Scale bar, 10 μm

As it is well acknowledged that *T. gondii* mature bradyzoite and complete cyst biogenesis cannot be obtained in vitro, we next examined the localization of BCLA in mature cysts isolated from mouse brain. WT-derived cysts—analyzed in vivo or ex vivo*—*showed a selective BCLA in situ staining, which delineated the cyst periphery, thus overlapping with the DBA signal of the WT cyst envelope, and this held true even when the membrane permeabilization procedure was omitted (Fig. 5a, b). Although cysts still formed in the context of infection with Δ*bcla* tachyzoites (Fig. 5b), yet they appeared smaller (Fig. 5c) and enclosed fewer bradyzoites, therefore showing a lower packing density, a feature reported [12], and an overall decline in GFP fluorescence (Fig. 5d). Moreover, the cysts loaded with the *Bcla*-deficient parasites kept surface reactivity with the DBA (Fig. 5e), but they displayed striking deformations of their cyst wall surface, as visualized by the loss of their circularity, and in some case by peculiar budding and segmented contours (Fig. 5f). To then assess whether these shape changes could weaken the cyst mechanical resistance, a phenotype previously reported for brain *Δcst1*-containing cysts [15], the cysts were subjected to mechanical stress post extraction from the brain tissue and isopycnic centrifugation-based purification (see the “Methods” section). No signs of increased susceptibility to these treatments were observed for cysts loaded with Δ*bcla* parasites except for occasional rupture. Overall, these data suggest that BCLA likely contributes to the cyst peripheral architecture and argue for its potential as a bona fide *T. gondii* cyst-specific biomarker. In this regard, BCLA/MAG2 was associated with both insoluble and soluble cyst matrix materials, suggesting that it interacts with the intracyst network (ICN) [18].

### BCLA is dispensable for efficient oral infection by bradyzoite-containing cysts

Being cyst specific, BCLA appeared dispensable for the cyst biology, since when orally delivered to C56BL/6 mice, the Δ*bcla* bradyzoite-loaded cysts remained fully infectious, inducing typical remodeling in intestinal crypt-villus (Fig. 4c, d) while in vitro Δ*bcla* tachyzoites showed no fitness loss (Fig. 4e). Consistently, the paired production of the IFNγ cytokine and the CCL2 chemokine in response to infection remained unchanged, when compared to mice receiving WT bradyzoite-loaded cysts (Fig. 4f). We next orally infected NMRI mice with 20 cysts of type II (76K) strains to evaluate the ability of Δ*bcla* parasites to disseminate via the bloodstream and settle in deep tissues where they differentiate into encysted bradyzoites. All the orally infected mice showed signs of illness with a substantial drop in weight over the acute phase of infection and they subsequently seroconverted. While cysts loaded of *Bcla*-deficient bradyzoites were infectious when delivered intragastrically in mice and promoted the development of a chronic infection, a small, yet not significant decrease in the brain cyst burden, characterized NMRI mice when compared to the BCLA^+^ cyst counterparts (Fig. 4g).

### Recombinant expression and purification of rBCLA for serological diagnosis

Given that the *blca* gene was exclusively expressed in bradyzoites and the BCLA protein was routed to the cyst, we investigated whether BCLA was immunogenic, a prerequisite for a serological cyst marker. Analysis and structure prediction of the BCLA sequence indicate a 140-kDa protein with an N-terminal signal peptide and a conserved C-terminal region of ~ 150 residues, with these two bordering a central core domain made of a motif of 48 amino acids repeated 13 times (Fig. 1d). Since the BCLA C-terminal region (aa 1089 to 1275) is predicted as the only ordered domain, we generated a recombinant C-terminal domain truncate fused to a poly-histidine tag using *E. coli* expression system, and hereafter referred to as rBCLA. With a theoretical molecular weight of 20.9 kDa and a pI of 4.7, rBCLA was subsequently purified through a multi-step chromatography workflow that included metal affinity (Additional file 3: Figure S2a), mono Q anion exchange (Additional file 3: Figure S2b), and size exclusion (Additional file 3: Figure S2c) chromatography, a combinatorial strategy which allowed for the first assessments of BCLA immunogenicity.

### The C-terminal part of BCLA is immunogenic and drives a specific humoral response in mice only over the chronic phase of infection by cystogenic *T. gondii* strains

**Fig. 6:**
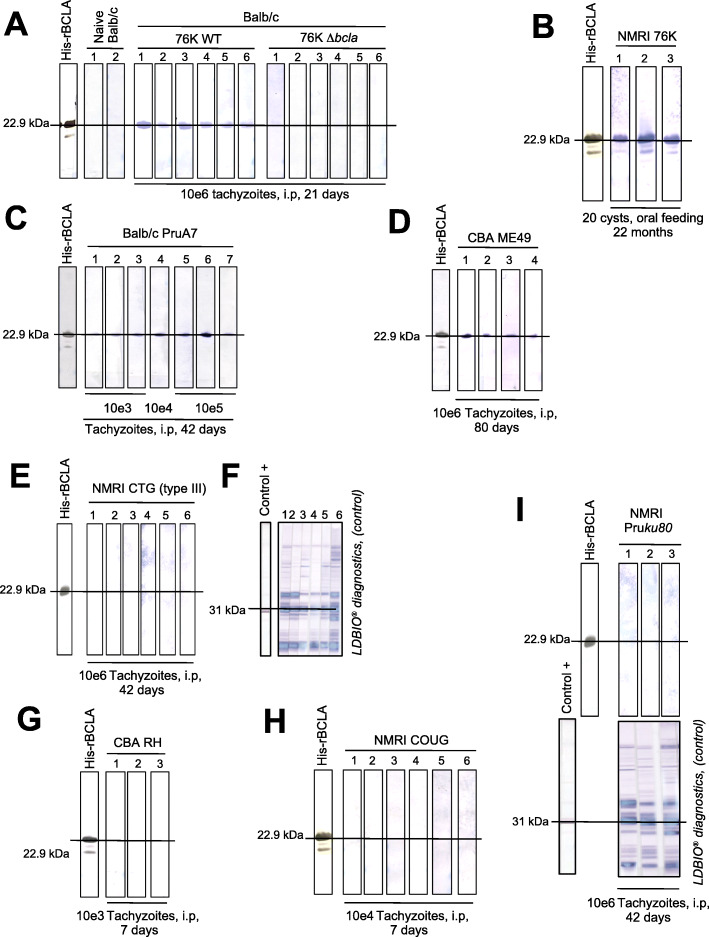
The C-terminal part of BCLA is immunogenic and drives a specific humoral response in mice only over the chronic phase of infection by cystogenic *T. gondii* strains. Single Western blot strips were loaded with 0.5 μg of recombinant rBCLA. The strips were tested on the sera collected from mice in the acute and chronic phases of toxoplasmosis with various *T. gondii* strains, route of infection, and mouse genetic background. **a** IgG immunoblot showing reactions of mouse antisera to rBCLA purified antigen. The sera were collected from BALB/c mice 21 days post-intraperitoneal inoculation of 10^6^ tachyzoites of 76K-GFP-luc WT or Δ*bcla* strains. **b** The sera were collected from NMRI mice 22 months post-orally inoculation of 20 cysts of the type II (76K-GFP-luc) strain. **c** The sera were collected from BALB/c mice 42 days post-intraperitoneal inoculation of 10^3^ to 10^5^ tachyzoites of the type II (PruA7) strain. The sera are reacting quite proportionally with rBCLA according to the tachyzoite load. **d** The sera were collected from CBA mice 80 days post-intraperitoneal inoculation of 10^6^ tachyzoites of the type II (ME49) strain. **e**, **f** IgG immunoblot showing reactions of mouse antisera to rBCLA purified antigen (**e**). The sera were collected from NMRI mice 42 days post-intraperitoneal inoculation of 10^6^ type III (CTG) tachyzoites. The serological status of the infected mice was determined by immunoblot using the LDBio Diagnostics test (**f**). **g** The sera were collected from CBA mice 7 days post-intraperitoneal inoculation of 10^3^ tachyzoites of the type I (RH) strain. **h** The sera were collected from NMRI mice 7 days post-intraperitoneal inoculation of 10^4^ tachyzoites of the haplotype 11 (COUG) strain. **i** The sera were collected from NMRI mice 42 days post-intraperitoneal inoculation of 10^6^ tachyzoites of the attenuated type II (PruΔ*ku80*). The serological status of the infected mice was determined by immunoblot using the LDBio Diagnostics test (lower panels)

When the SDS-PAGE resolved rBCLA and tachyzoite-specific antigens (LDBio [25];) were probed by Western blot using the sera from NMRI and BALB/c mice collected post-injection of either WT or Δ*bcla* 76K inocula and at different stages of toxoplasmosis, the selective rBCLA reactivity unambiguously documented a sustained production of anti-BCLA antibodies during the chronic phase of infection (Fig. 6). Indeed, no signal was detected using the sera collected from naive or Δ*bcla* tachyzoite-infected BALB/c mice whereas 100% of the mice inoculated with WT 76K tachyzoites (Fig. 6a) or orally fed with 76K cysts (Fig. 6b) developed anti-BCLA antibodies within 21 days post-infection (Fig. 6a), a period compatible with early cystogenesis and their production was shown to persist over months (Fig. 6b). Similarly, all the sera from BALB/c and CBA mice injected with tachyzoites from other cystogenic strains, namely PruA7 and ME49, reacted positively with rBCLA at times exceeding 40 days post-inoculation (Fig. 6c, d). In contrast, the sera from mice inoculated with tachyzoites of the CTG strain, for which BCLA antigen remained undetectable (Fig. 3c), failed to react against rBCLA (Fig. 6e) despite the mice showing seroconversion for tachyzoite antigens, in particular SAG1 (Fig. 6f). This was equally true for non-cystogenic -RH type I- or notoriously poorly cystogenic - non-canonical COUG [26] and type II culture-attenuated PruΔ*Ku80* strains-, regardless of the hosting mice’ genetic background (Fig. 6g–i). Furthermore, the sera from mice fed with cysts and collected during the acute phase of infection, hence prior to cystogenesis, showed no rBCLA reactivity (Additional file 4: Figure S3a), whereas corticoid-based immunosuppressive therapy of chronically infected mice, which led to massive tachyzoite proliferation, did not enhance the anti-rBCLA antibody production (Additional file 4: Figure S3b).

### rBCLA is accurately detected by ELISA in sera of mice bearing cysts

**Fig. 7:**
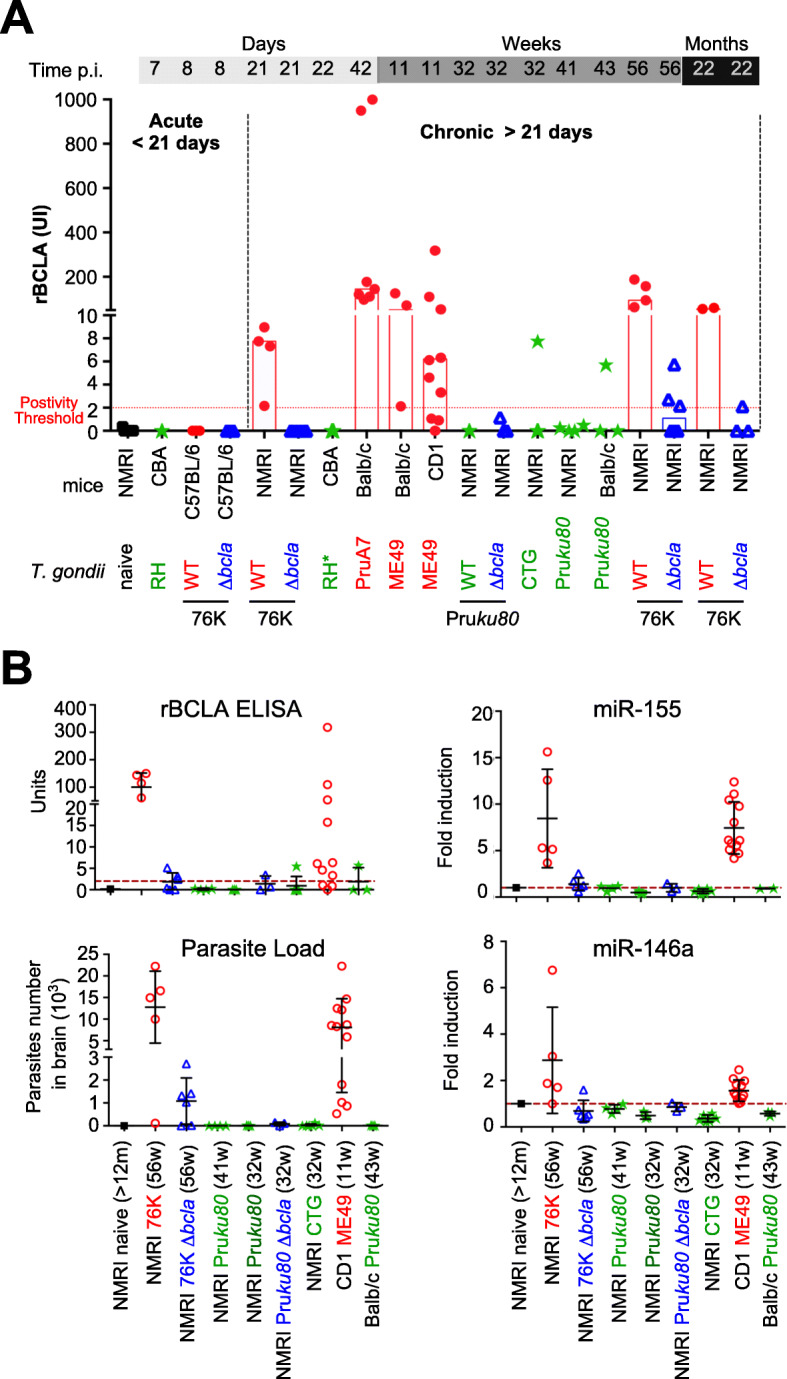
ELISA-based serological analyses of rBCLA presence in the sera of mice. **a** ELISA serological titration of rBCLA reactivity in mice over time and for a given *T. gondii* strain. Individual ELISA measurements given in UI are grouped according to *T. gondii* strain type with cystogenic strains (ME49, PruA7, 76K; red spots), the non-cystogenic strain RH (green stars), the culture-attenuated strains (Pru*ku80*, CTG; green stars), and Δ*bcla* strains (in 76K or Pru*ku80* backgrounds; blue triangles). A time segmentation post-infection is displayed to distinguish between the acute phase (≤ 8 days) and chronic phase (≥ 21 days). **b** rBCLA ELISA reactivity correlation with parasitic load, miR-155, and miR-146a expression. Superposed titrations of rBCLA IgGs (in UI), parasitic load (in parasite qPCR count), and miR-155/miR-146a are shown for different mouse strains (NMRI, BALB/c), non-infected or infected with different *T. gondii* strains all within a chronic infection timeframe (≥ 11 weeks). Cystogenic strains (ME49, PruA7, 76K; red circles), the non-cystogenic strain RH (green stars), the culture-attenuated strains (Pru*ku80*, CTG; green stars), and Δ*bcla* strains (in 76K or Pru*ku80* backgrounds; blue triangles) were used

Higher accuracy serum antibody assays were further achieved by developing an indirect ELISA test using rBCLA (see the “Methods” section). Overall, the semi-quantitative analysis of anti-rBCLA antibody titers identified BALB/c, CD1, and NMRI mice that were likely bearing WT cysts, as highly responsive to BCLA with an increased yield over time (Fig. 7a). The rBCLA detection correlated well with the presence of fully developed cysts in the brain (> 21 days p.i.) that persisted throughout the life of the chronically infected mice (up to 22 months p.i.). In contrast, the sera of naive mice or infected with Δ*bcla* were negative as were those of mice infected with strains known as low-cyst producers (e.g., culture-attenuated PruΔ*Ku80* and CTG) (Fig. 7a). By combining data of quantitative PCR conducted on DNA of brain-associated *T. gondii* DNA with the quantitation of brain-associated miR-155 and miR-146 host microRNAs that were reported to be specifically induced upon bradygenesis [27], we brought additional evidence of the antigenic feature of rBCLA and its usefulness to serologically assess the presence of *T. gondii* bradyzoite-loaded cysts over the long-lasting protozoan persistence in rodents (Fig. 7b). Collectively, these data also allowed designating the BCLA antigen as the “brain cyst load-associated antigen”.

### Epitope mapping in rBCLA positive patients reveals a multitude of antigenic regions within rBCLA and consistent reactivity within the repeated region

**Fig. 8:**
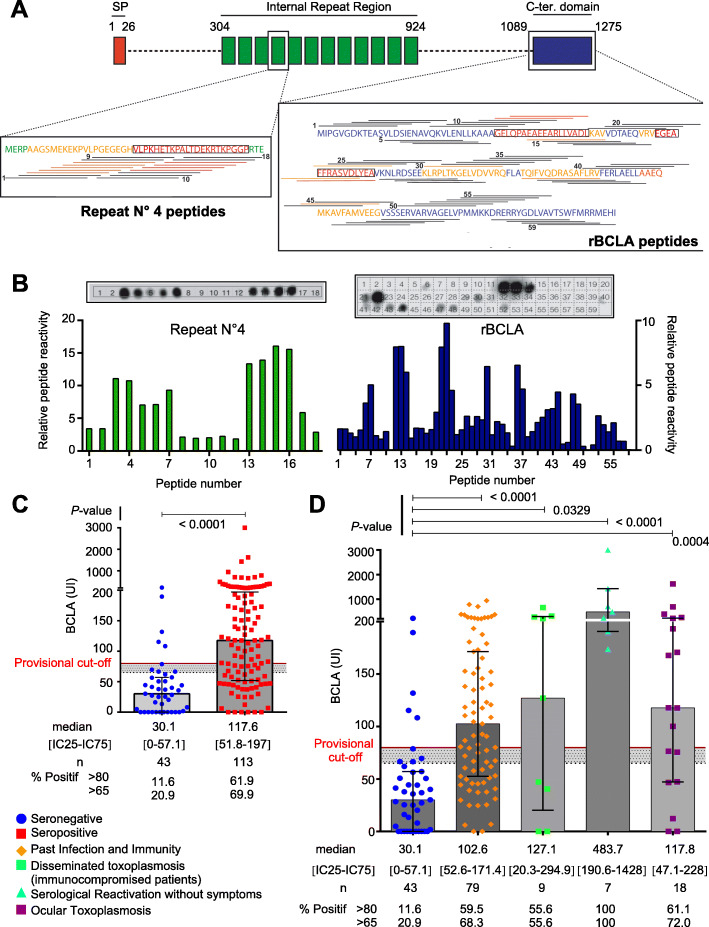
BCLA behaves as a bona fide serological marker for chronic infection in humans. **a** Schematic representation of the epitope-mapped regions in both the repeat n°4 and rBCLA region. Peptide coverage is displayed as a line representing the individual 14aa peptides above or below the peptidic sequence, with partial numbering displayed. Regions displaying significant or strong reactivity are colored orange and red, respectively. **b** Epitope mapping of the sera positive by Western blot to rBCLA. Below, histograms displaying the relative reactivity of peptides on both the core repeat region and rBCLA region, calculated using 5 different blots from sera Western blot reactive to rBCLA with a negative non-specific background subtraction of 5 individual sera negative by Western blot to rBCLA and also Sag1 negative. Above is an example of a dot blot membrane revelation pattern with numbered peptides, performed on a positive human serum. **c** BCLA reactivity in human sera depending on *T. gondii* serological status. Scatter plot of individual BCLA ELISA titrations (in UI) in seronegative (blue dots) and seropositive individuals (red squares) assessed through routine clinical serologies against *T. gondii* infection (Vidas® and Architect® IgG/IgM). Gray histograms display the median BCLA titration value per group and interquartile range. Statistical significance was calculated using a non-parametric Mann-Whitney test. Gray zone (65 to 80 UI) and positivity cutoff line (at 80 UI) are indicated. **d** Detailed BCLA reactivity within clinical categories. Scatter plot of individual BCLA ELISA titrations (in UI) grouped within clinical status categories of *T. gondii* seropositive patients compared to the seronegative group. These groups are as follows: SAG1 seronegative patients (blue dot), past immunity patients (orange diamonds), active toxoplasmosis in immunocompromised patients (green cubes), asymptomatic serological reactivation in immunocompromised patients (turquoise triangle), and proven ocular toxoplasmosis patients (purple cube). Gray histograms display the median BCLA titration value per group and interquartile range. Statistical significance was calculated using the Kruskal-Wallis non-parametric test followed by Dunn’s post-test to compare individually all the latter groups with the seronegative patients’ groups. Gray zone (65 to 80 UI) and positivity cutoff line (at 80 UI) are indicated

Whether BCLA could be validated as a marker of *T. gondii* cysts in humans was the next most obvious challenge. Relying on the toxoplasmosis biobank for providing human sera of interest, we optimized the ELISA test for the detection of BCLA immunogenic peptides. First, peptide microarrays designed using both the BCLA C-terminal domain and the most conserved internal peptide repeat sequences (Fig. 8a) were screened for high-resolution BCLA epitope mapping (Fig. 8b and Additional file 5: Figure S4). These arrays were conceived using 15 aa peptides with a 3 aa gap step between peptides (Fig. 8a). We then tested several sera which were unequivocally positive by Western blot against rBCLA and taking the same number of negative sera to proportionally subtract non-specific reactivity. When analyzed, the total reactivity score for each peptide obtained on both the repeat region and rBCLA (Fig. 8b) provided us two key information: first, rBCLA despite having several stronger zones of reactivity, notably close to peptides 13, 22, 30, and 43, has a multitude of epitopes throughout the domain and different sera react quite differently to different zones (Additional file 5: Figure S4b). This underlines the requirement for keeping the rBCLA as a recombinant protein within the ELISA test. Moreover, as the rBCLA domain is predicted as structured, structured epitopes would only be provided by the presence of recombinantly expressed and properly folded protein, further underlining its use. Second, the repeat motif is found to consistently react in two separate zones (peptides 3 to 7 as motif A and 13 to 16 as motif B) in all the 5 sera tested previously positive to rBCLA by Western blot. This feature underscores the need to include one or several peptides covering these motifs to increase the ELISA sensitivity. Although the first generation of ELISA test was originally designed with rBCLA, the discovery of the antigenic repeat region by dot blot led us to screen the sera by ELISA for chemically synthetic peptides from which we found ABf and A3b to be the most specifically reactive out of 10 tested variants in length and sequence. These two peptides could not be used as ELISA antigens on their own as the overall reactivity of the test was reduced, but once combined with rBCLA, the peptides improved the reactivity of the assay for human sera (Additional file 6: Figure S5) and were subsequently included for both human and mouse assays.

From these results, we therefore designed an ELISA which combines rBCLA recombinant protein and chemically synthetized repeat motifs. The first ELISA test was optimized for the detection of rBCLA immunogenic IgGs. However, in contrast to mice, all positive human sera showed a robust reactivity to peptides derived from the internal repeat that significantly increased the test sensitivity, once added to rBCLA. Therefore, the BCLA ELISA was customized based on the most sensitive peptide and polypeptide combination. When assessing BCLA in comparison with a standard *T. gondii* serology status, BCLA titers are significantly increased in *T. gondii* seropositive sera but also show a widespread distribution with some sera not reactive to BCLA within that group (Fig. 8c, Table 1 and Additional file 7: Table S2). When we segment *T. gondii*-positive patients, by clinical category, anti-BCLA IgG titers are significantly higher in the subpopulations of patients with pathologies strongly linked to the presence of cysts. In particular, this is prominent in patients with serological reactivation in the context of immunosuppression and in those with established ocular toxoplasmosis. For a few patients diagnosed with ocular toxoplasmosis, we were able to detect BCLA antibodies by ELISA both in their aqueous humor and in their serum. The ELISA test also detected significant amounts of circulating anti-BCLA antibodies in the sera from immunocompromised patients undergoing either “asymptomatic” or “symptomatic” toxoplasmosis following cystic reactivation (Fig. 8d, Table 1, and Additional file 7: Table S2). Some of the highest titration are recorded in these groups, but again, the distribution is spread out as non-reactive sera are also detected in these groups.

**Table 1:** Titration of BCLA in different clinical forms of toxoplasmosis

	BCLA [UI]	IgG [IU/mL] (Architect)	IgM [index] (Architect)	IgG [IU/mL] (Vidas)	IgM [index] (Vidas)	Clinical context
Serological interpretation						
Negative	< 65	< 1.6	< 0.5	< 4	< 0.55	
Inconclusive	65–79	1.6–2.9	0.50–0.59	4–7	0.55–0.64	
Positive	≥ 80	≥ 3	≥ 0.6	≥ 8	≥ 0.65	
*Toxoplasma* seronegative patients (*n* = 43)						
*Median [IC25–IC75]*	30.1 [0–57.1]	0.1 [0–0.1]	0.09 [0.07–0.14]	/	/	
*Patient 1*	0	0.2	0.14	/	/	Immunocompetent/delivery
*Patient 2*	37.8	0.3	0.09	/	/	Immunocompetent/corneal transplant
*Patient 3*	115.3	0.1	0.07	/	/	Immunocompetent/otitis
*Patient 4*	69.6	0.2	0.12	/	/	Immunosupression/AIDS
*Patient 5*	16.1	0.1	0.06	/	/	Immunocompetent/pregnancy
Past immunity—chronic toxoplasmosis (*n* = 79)						
*Median [IC25–IC75]*	102.6 [52.6–171.4]	29.7 [7.95–69.9]	0.19 [0.10–0.31]	132 [41–252]	0.07 [0.04–0.24]	
*Patient 6*	61.8	22.9	0.10	101	0.03	Immunocompetent/pregnancy
*Patient 7*	118.4	523	0.68	> 300	0.66	Immunocompetent/pregnancy
*Patient 8*	180.5	26.3	0.30	120	0.27	Immunosupression/dysglobulinemia
*Patient 9*	264.9	31.6	0.41	164	0.26	Immunocompetent/pregnancy
*Patient 10*	77.2	68.3	0.11	199	0.11	Immunocompetent/living kidney donor
Disseminated toxoplasmosis in immunocompromised patients (*n* = 9)						
*Median [IC25–IC75]*	127.1 [20.3–294.9]	28.4 [1.4–269.9]	0.11 [0.08–0.13]	42 [2.5–300]	0.10 [0.04–0.21]	
*Patient 11*	0	1.3	0.12	2	0.24	Allogeneic HSCT/disseminated toxoplasmosis/positives PCR in BAF, buffy coat and CSF
*Patient 12*	40.5	/	/	42	0.17	Allogeneic HSCT/cerebral toxoplasmosis/positives PCR in buffy coat and CSF
*Patient 13*	277.4	78.8	0.11	> 300	0.11	AIDS/cerebral toxoplasmosis/positives PCR in buffy coat and CSF
*Patient 14*	127.1	/	/	> 300	0.1	AIDS/cerebral toxoplasmosis/positive PCR in CSF
*Patient 15*	213.3	28.4	0.09	25	0.03	Neoplasia/pulmonary toxoplasmosis/positive PCR in BAF
Asymptomatic serological reactivation in immunocompromised patients (*n* = 7)						
*Median [IC25–IC75]*	483.7 [190.6–1427.8]	359.0 [54.7–1254]	0.86 [0.26–4.93]	300 [26–300]	1.33 [0.22–5.26]	
*Patient 16*	235.1	1254	0.86	> 300	1.33	Allogeneic HSCT/3 negative PCR in buffy coat
*Patient 17*	701.8	936.4	0.26	> 300	0.04	Allogeneic HSCT
*Patient 18*	1427.8	359.0	2.68	> 300	2.76	Allogeneic HSCT/2 negative PCR in buffy coat
*Patient 19*	173.8	> 2000	0.17	> 300	0.26	Allogeneic HSCT
*Patient 20*	190.6	107.1	6.18	23	5.26	Allogeneic HSCT/2 negative PCR in buffy coat
Ocular toxoplasmosis (*n* = 18)						
*Median [IC25–IC75]*	117.7 [47.1–227.9]	87.7 [25.6–405.5]	0.21 [0.09–1.53]	199 [135–300]	0.09 [0.04–0.86]	
*Patient 21*	46.6	/	/	264	0.10	Immunosuppression/positive PCR in AH
*Patient 22*	167.7	71.5	0.22	178	0.14	Immunocompetent/positive PCR in AH
*Patient 23*	1622.2	> 2000	0.15	> 300	0.11	Immunocompetent/positive PCR in AH
*Patient 24*	75.5	11	0.09	48	0.04	Immunocompetent/production of antibodies in HA
*Patient 25*	193	370.8	9.01	209	4.99	Immunocompetent/production of antibodies in HA

*Toxoplasma* IgG, IgM levels, and clinical context of five patients per clinical forms of toxoplasmosis are described (for all patients of this study cf. supplemental data)

*HSCT* hematopoietic stem cell transplantation, *AIDS* acquired immunodeficiency syndrome, *BAF* broncho-alveolar fluid, *CSF* cerebro-spinal fluid, *AH* aqueous humor

## Discussion

Biomarker discoveries are a major challenge for improving disease management, in particular, in the context of toxoplasmosis recurrence that follows the reactivation of encysted parasites commonly persisting in the nervous system or muscle tissues. The most prevailing view is that *Toxoplasma* infection provides forever protection, and this possibly because of the continuous stimulation of the immune system by antigens released from the cysts [28]. Yet reliable specific serological markers of tissue cysts were heretofore missing for both research and medical diagnosis purposes. This study starts to fill the gap by identifying the *bcla* transcript and the BCLA/MAG2 protein as bradyzoite-specific, with BCLA/MAG2 eventually localized at the periphery of the cyst loaded with *T. gondii* bradyzoites. Of note, being restricted to the Coccidia subclass of *Apicomplexa* (www.ToxoDB.org data), the *bcla* gene showed only low level of homology even between the closest members of the subclass; thus, *T. gondii* BCLA/MAG2 could be positioned as a valuable biomarker candidate. Finally, serological Western blot and ELISA assays confirmed a *T. gondii* BCLA-driven humoral response in rodents and also indicated that this hold held true for humans, therefore providing BCLA with the appropriate features for a cyst-specific antigenic marker.

The conclusions have however several limitations and raised questions. First, a non-negligible amount of sera appears as outliers within their respective groups. For instance, there is a current discrepancy between SAG1 negative serologies and BCLA serologies which still display a false apparent positive discovery rate of around 12% (based on an 80 UI cutoff). This could be explained in some sera by non-specific interactions with the different BCLA epitopes as some of these patients suffer from recurring viral infection and other chronic diseases susceptible to produce non-specific cross-reactive antibodies. Another possibility is the presence of immunogenic exogenous bacterial contaminants that likely co-purify with rBCLA, and this problem could be addressed by designing different expression/purification protocols. Finally, there may also potentially be truly BCLA-positive patients with negative SAG1 serologies. The second main observation is that some clinical profiles within the *T. gondii* seropositive group, most notably the “asymptomatic serological reactivation in immunocompromised patient” group, had a tendency to generate vastly stronger immunogenic reactions where BCLA serologies were titrated well beyond the median positive BCLA serology in the “past immunity” group. Finally, for some groups where a BCLA positivity would always be expected, such as in the case of “active toxoplasmosis in immunocompromised patient” and “ocular toxoplasmosis,” only a minority of serologies still remained poorly reactive. These serology results can highlight the limitation of sensitivity from the ELISA test or potentially illustrate that BCLA serologies can turn negative during immunosuppression as many of these patients within these groups actually were undergoing immunosuppression (Additional file 7: Table S2).

Remarkably, while IgM and IgA antibody isotypes are frequently associated with *T. gondii* primary and acute infection, a persistent and steady-state IgG level and absence of IgM rather characterize past and latent infections [29]. We have only detected anti-BCLA IgG isotype and this, exclusively over the course of chronic infection. Contrasting with the IgM response reported in mice 10 days after oral ingestion of cysts [30], the IgG isotype restriction of anti-BCLA antibodies reinforces the view of BCLA as a timely marker for the latent stage of *T. gondii* human and animal infections. Therefore, rBCLA fills the criteria required for a serologic predictive marker of cystogenic potential in clinical isolates.

## Conclusions

Being validated as a specific serological marker for the presence of persistent *T. gondii* parasites, BCLA/MAG2 could in the long term assist in the design of a combinatorial serology-based strategy to guide transplantation medicine and immunotherapy protocols. In the context of clinical research, BCLA/MAG2 could represent a primary asset to revisit the dogma of life-long cyst persistence as a driver of immune-based protection against reinfection. This dogma is currently challenged by the reduction of seroprevalence in population worldwide and the identification of *T. gondii* reinfection in immunocompetent people [28], but it was not being properly assessed in the absence of specific serological cyst markers. The BCLA-based marker should prove useful at refining the epidemiological correlation between chronic infection and neuropsychiatric symptoms. Finally, this marker could also allow the monitoring of the burden of *T. gondii* infection in HIV-infected population, an important issue for the improvement of routine surveillance, especially in sub-Saharan African country where 87% of global cases of co-infection were recently reported [31].

## Methods

### Parasites and human cell culture

Human primary fibroblasts (HFFs, ATCC® CCL-171™) were cultured in Dulbecco’s modified Eagle’s medium (DMEM) (Invitrogen) supplemented with 10% heat-inactivated fetal bovine serum (FBS) (Invitrogen), 10 mM (4-(2-hydroxyethyl)-1-piperazine ethanesulphonic acid) (HEPES) buffer pH 7.2, 2 mM l-glutamine, and 50 μg/mL of penicillin and streptomycin (Invitrogen). Cells were incubated at 37 °C in 5% CO_2_. The *Toxoplasma* strains used in this study and listed in Additional file 2: Table S1 were maintained in vitro by serial passage on monolayers of HFFs. The cultures were free of *Mycoplasma*, as determined by qualitative PCR.

### Reagents

The following primary antibodies were used in the immunofluorescence, immunoblotting, and/or ChIP assays: rabbit anti-TgHDAC3 (RRID: AB_2713903), rabbit anti-H4K31ac (RRID: AB_2811024), rabbit anti-H4K31me1 (RRID: AB_2811025), rat anti-CC2 (gift from W. Bohne and U. Gross), mouse anti-TgBAG1, mouse anti-HA tag (Roche, RRID: AB_2314622), rabbit anti-HA Tag (Cell Signaling Technology, RRID: AB_1549585), rabbit anti-acetyl-Histone H4, and pan (Lys 5,8,12) (Millipore, RRID:AB_310270). Immunofluorescence secondary antibodies were coupled with Alexa Fluor 488 or Alexa Fluor 594 (Thermo Fisher Scientific). Secondary antibodies used in Western blotting were conjugated to alkaline phosphatase (Promega) or horseradish peroxidase. We also raised homemade BCLA-specific antibodies in rabbit against linear peptides of BCLA, i.e., peptide 1: C-EMERPAAGSMEK and peptide 2: C-VLPKHETKPALT. They were produced by Eurogentec and used for immunofluorescence and immunoblotting.

### Mouse infection and experimental survey

Six-week-old BALB/c, CBA, NMRI, C57BL/6, or Swiss mice were obtained from Janvier Laboratories (Le Genest-Saint-Isle, France). Mouse care and experimental procedures were performed under pathogen-free conditions in accordance with established institutional guidance and approved protocols from the Institutional Animal Care and Use Committee of the University Grenoble Alpes (agreement n°B3851610006). Female mice were used for all studies. For intraperitoneal (i.p.) infection; tachyzoites were grown in vitro and extracted from host cells by passage through a 27-gauge needle, washed three times in phosphate-buffered saline (PBS), and quantified with a hemocytometer. Parasites were diluted in Hank’s Balanced Salt Solution (Life), and mice were inoculated by the i.p. route with tachyzoites of each strain (in 200 μl volume) using a 28-gauge needle. For oral gavage of infectious cysts, the brains from chronically infected mice (76K-GFP-luc and 76K-GFP-luc-Δ*bcla*) were crushed in PBS, the number of cysts was microscopically quantified, and the mice were forced fed with 100 μl of brain homogenate containing 20 to 40 cysts using ball-tipped feeding needle. Blood was collected by caudal puncture or by intracardiac puncture when the mice were euthanized. Animal euthanasia was completed in an approved CO_2_ chamber. For histological analysis of ileum and immunolabeling on histological sections of the brains, the ilea and brains were removed from mice, entirely embedded in a paraffin wax block and cut in 5-μm-thick layers using microtome. For statistical analysis of mouse survival data, the Mantel-Cox and Gehan-Breslow-Wilcoxon tests were used.

### Auxin-induced degradation

Depletion of MORC-AID-HA was achieved with 3-indoleacetic acid (IAA, Sigma-Aldrich # 45533) used at 500 μM final concentration from a 500-mM stock solution prepared in EtOH. To monitor the degradation of AID-tagged proteins, parasites grown in HFF monolayers were exposed to auxin, or the ethanol vehicle alone, for different time intervals at 37 °C before parasites were harvested and analyzed by immunofluorescence or Western blotting.

### HDAC3 inhibition using FR235222

FR235222 was provided by Astellas Pharma Inc. (Osaka, Japan) and dissolved into DMSO, and the final concentration in the culture medium was either 25 or 50 ng/mL. Sixteen hours after infection of HFF monolayers, FR235222 was added to the medium and cells cultivated for 24 h to 7 more days.

### Immunofluorescence microscopy

*T. gondii*-infected HFF monolayers grown or cysts purified from mouse’s brains were fixed in 3% formaldehyde for 20 min at room temperature, permeabilized with 0.1% (v/v) Triton X-100 for 15 min, and blocked in PBS containing 3% (w/v) bovine serum albumin (BSA). For immunolabeling on histological sections of the brains, the brain layers spotted on glass slides were first solvent-dewaxed using toluene for 3 times 10 min and absolute alcohol for 3 times 10 min. The slides were then treated with citrate buffer pH 6, heated at 100 °C during 1 h, rinsed extensively with water and blocked in PBS containing 3% (v/v) BSA. The infected cells or brain layers were then incubated for 1 h with the primary antibodies indicated in the figures followed by the addition of secondary antibodies conjugated to Alexa Fluor 488 or 594 (Molecular Probes) at a 1:1000 dilution for 1 h. The nuclei of both host cells and parasites were stained for 10 min at room temperature with Hoechst 33258 at 2 μg/mL in PBS. After four washes in PBS, coverslips were mounted on a glass slide with Mowiol mounting medium; images were acquired with a fluorescence ZEISS ApoTome.2 microscope and processed with the ZEN software (Zeiss).

### Western blot

Immunoblot analysis of protein was performed as described [32]. Briefly ~ 10^7^ cells were lysed in 50 μl lysis buffer (10 mM Tris-HCl, pH 6.8, 0.5% SDS [v/v], 10% glycerol [v/v], and 1 mM EDTA and protease inhibitors cocktail) and sonicated. Proteins were separated by SDS-PAGE and transferred to a polyvinylidene fluoride membrane (PVDF, Immobilon-P; EMD Millipore) by liquid transfer 1h30 at 100 V; blotted membranes were then probed using appropriate primary antibodies followed by alkaline phosphatase or horseradish peroxidase-conjugated goat secondary antibodies (Life technologies). Band revelation was detected using NBT-BCIP (Amresco) or enhanced chemiluminescence system (Thermo Scientific).

### Cyst purification

Cysts were isolated from the brains of mice chronically infected with the 76k-GFP-luc or the 76k-GFP-luc-Δ*bcla* strains for at least 6 weeks, either using the Percoll gradient method as described previously [33] either directly harvesting the cysts using a 10-μl pipet for the dyes experimentation in order not to deteriorate the cyst wall for permeability studies. Neither saponin nor trypsin was added at the end of the experiment.

### Cyst quantification

Five to 12 weeks post-infection, the brain of each of the recipient mouse was homogenized in 2 mL of PBS. The numbers of cysts in three or ten aliquots (200 μl each) of the brain suspensions were counted microscopically. For statistical analysis of cyst quantification differences between mice infected with 76K-GFP-luc and 76K-GFP-luc-Δ*bcla*, the non-parametric Wilcoxon-Mann-Whitney test was applied.

### Cyst characterization

Images of semi-purified cysts were acquired between slide and slip cover with a fluorescence ZEISS ApoTome.2 microscope. Cysts areas and GFP signals were quantified using the ZEN software (Zeiss). For statistical analysis of cysts areas and GFP-fluorescence differences between 76K-GFP-luc and 76K-GFP-luc-Δ*bcla* cysts, the non-parametric Wilcoxon-Mann-Whitney test was applied.

### Histological analysis of ileum

Fragments of the small intestines of mice infected with WT or *bcla*-deficient cysts were harvested on day 8 post-infection, fixed in 10% buffered formalin, and paraffin processed. Tissue sections that were 5-μm-thick were mounted on slides and stained with hematoxylin and eosin. The histological score was analyzed with the following parameters: intensity of the lamina propria inflammatory infiltration, thickening of lamina propria, and destruction of the villi and necrosis, which were evaluated by intensity, represented as arbitrary units ranging from zero (less intense or absent) to six (highly intense) for each parameter.

### Quantitative PCR

The parasite loads in the brain or ileum were quantified following DNA extraction (QiAmp DNA mini kit, Qiagen) using the quantitative PCR targeting of the *Toxoplasma*-specific 529-bp repeat element [34]. For statistical analysis of parasitic load differences between mice infected with 76K-GFP-luc and 76K-GFP-luc-Δ*bcla*, the non-parametric Wilcoxon-Mann-Whitney test was applied.

### Quantitative RT-PCR analysis of interleukins in the brain and ileum

Total RNA was isolated from brains or ilea using TRIzol (Thermo Fisher Scientific). cDNA was synthesized with random hexamers by using the high Capacity RNA-to-cDNA kit (Applied Biosystems). Samples were analyzed by real-time quantitative PCR for appropriate probes (brain: TNF-α, INF-γ, IL-6, and IL-22β; ileum: INF-γ, CCL2, IL-22β, IL-18, and IL-1β) using the TaqMan Gene Expression Master Mix (Applied Biosystems). RNA levels were normalized using TBP levels. Quantitative RT-PCR was repeated for three independent biological replicates of each sample. For statistical analysis of RNA levels between mice infected with 76K-GFP-luc and 76K-GFP-luc-Δ*bcla*, the non-parametric Wilcoxon-Mann-Whitney test was applied.

### DBA lectin labeling on in vitro FR235222 parasites and ex vivo cysts

*T. gondii*-infected HFF cells grown on coverslips or cysts purified from the brains of mice were fixed in 3% formaldehyde for 20 min at room temperature, permeabilized with 0.1% (v/v) Triton X-100 for 15 min and blocked in PBS containing 3% (v/v) BSA. The infected cells or cysts were stained with 1:100-diluted Dolichos lectin for 30 min. The stained vacuoles or cysts were examined with a fluorescence ZEISS ApoTome.2 microscope, and images were processed by the ZEN software (Zeiss).

### *Toxoplasma gondii* transfection

*T. gondii* strains were electroporated with vectors in cytomix buffer (120 mM KCl, 0.15 mM CaCl_2_, 10 mM K_2_HPO_4_/KH_2_PO_4_ pH 7.6, 25 mM HEPES pH 7.6, 2 mM EGTA, 5 mM MgCl_2_) using a BTX ECM 630 machine (Harvard Apparatus). Electroporation was performed in a 2 mm cuvette at 1.100 V, 25 Ω and 25 μF. When needed, the antibiotic (concentration) used for drug selection was chloramphenicol (20 μM), mycophenolic acid (25 μg/mL) with xanthine (50 μg/mL), pyrimethamine (3 μM), or 5-fluorodeoxyuracil (10 μM). Single-clone of stable transgenic tachyzoites were obtained by limiting dilution in 96-well plates and verified by immunofluorescence assay or genomic analysis.

### Plasmid construction

The plasmids and primers for the genes of interest (GOI) used in this work are listed in Additional file 2: Table S1. To construct the vector pLIC-GOI-HAFlag, the coding sequence of GOI was amplified using primers LIC-GOI-Fwd and LIC-GOI-Rev using *T. gondii* genomic DNA as template. The resulting PCR product was cloned into the pLIC-HF-dhfr or pLIC-mCherry-dhfr vectors using the ligation-independent cloning (LIC) cloning method [35]. The plasmid pTOXO_Cas9-CRISPR was described previously [36]. Twenty mer-oligonucleotides corresponding to specific GOI were cloned using the Golden Gate strategy. Briefly, primers GOI-gRNA-Fwd and GOI-gRNA-Rev containing the sgRNA targeting GOI genomic sequence were phosphorylated, annealed, and ligated into the pTOXO_Cas9-CRISPR plasmid linearized with BsaI, leading to pTOXO_Cas9-CRISPR::sgGOI.

### Recombinant expression of the C-terminal domain of BCLA (rBCLA)

#### Design and cloning

To recombinantly express the domain, the N-terminal boundary was chosen at the methionine 1089 and the original C-terminal end was conserved. DNA synthesis was performed by Genscript to generate a fusion construct composed of rBCLA (1089-1275) with a TEV cleavable N-terminal His-tag (Fig. 1d and Additional file 2: Table S1). Codon optimization for *E. coli* was performed, and the gene was cloned by Genscript within a pet30-(a) vector (Addgene) using NdeI and XhoI sites.

#### Recombinant expression

Transformation was performed using BL21(DE3)-CodonPlus—RIL chemically competent *E. coli* (Stratagene) which were incubated on ice with 1 μg of the pET30-(a) rBCLA plasmid for 10 min heat shocked at 42 °C for 45 s, pre-incubated 45 min in LB at 37 °C then spread on a LB agar plate containing kanamycin (Kan) and chloramphenicol (Chlo) and incubated for 12 h. A single colony was then picked to inoculate a LB/Kan/Chlo 50 mL pre-culture grown for 16 h; 5 mL of grown pre-culture was then used to inoculate 1-L flasks of Terrific Broth medium (Formedium) containing Chlo/Kan. Cultures were grown at 37 °C until reaching an OD_600_ of 0.5–0.8 then induced by adding 0.7 mM IPTG (VWR) and further incubated at 18 °C ON. After incubation, cells were centrifuged 25 min at 3000*g*, the supernatant was discarded, and the pellet flash-frozen in liquid nitrogen and kept at − 80 °C.

#### Bacteria lysis

Purification was performed on 3 pellets of 1 L culture, each resuspended in 50 mL of lysis buffer containing (600 mM NaCl, 50 mM Tris pH 8, 5 mM beta-mercaptoethanol (BME), 0.2% w/v *N*-Lauryl Sarkozine and 1 complete anti-protease cocktail (Roche) tab per 50 mL) and subjected to a 10-min pulsed sonication (15 s ON, 30 s OFF) at 50° amplitude over ice to remain below 13 °C. After sonication, the lysate was centrifuged at 4 °C for 1 h at 15,000*g*, and the pellet was discarded. All the following steps were subsequent at 4 °C. Prior to incubation with 5 mL of pre-equilibrated Ni-NTA resin, the clarified lysate was supplemented with 30 mM imidazol. Batch incubation was performed for 30 min at 4 °C with a gentle stirring. After incubation, the resin was retained on a vertical column then washed with 4 column volumes (CV) of wash buffer (600 mM NaCl, 50 mM Tris pH 8, 5 mM BME, 0.2% w/v *N*-Lauryl Sarkosine and 30 mM Imidazole). Elution was then performed stepwise by collecting fraction of 1.5 mL using the elution buffer (300 mM NaCl, 50 mM Tris pH 8, 5 mM BME, and 300 mM imidazole). Fractions of interest were then pooled and dialyzed in 50 mM NaCl, 50 mM Tris pH 8.5 mM BME using a 10-kDa cutoff dialysis cassette (Thermo Scientific).

#### Ion exchange and size exclusion chromatography

The entire sample was then directly pumped through the chromatography system (Akta Pure, GE healthcare) onto a HL-Mono-Q (GE healthcare) 5 mL column pre-equilibrated with the same buffer as for dialysis. The column was washed with 2 CV then eluted by a salt gradient (50 mM to 2 M NaCl) over 40 mL, 1.5 mL fractions and 280 nm absorbance monitoring was performed over the whole elution period. SDS PAGE analysis revealed that the sample was purified in the later stages of the gradient elution and that the early elution fractions presented most of the bacterial contaminants visible at higher molecular weight. Desired fractions were collected, pooled, and concentrated to 600 μl using a 10-kDa cutoff concentrator (Amicon-Ultra, Millipore). After concentration, the sample was injected on a S75 (GE healthcare) with a running buffer containing 150 mM NaCl, 50 mM Tris pH 8, and 5 mM BME and eluted in a heterogeneous peak consistent with a multimeric state, starting close to the void volume and eluting over 3 mL. All elution fractions were pooled to generate the final sample.

#### Ammonium sulfate precipitation

To avoid nucleic acid contamination, an ammonium precipitation was performed by adding 15% w/v of ammonium sulfate (Sigma), gentle rolling at 4 °C for 1 h then 30 min of centrifugation at 10,000*g*. The supernatant was discarded, and the pellet resuspended in the same initial volume of buffer. To clear all ammonium sulfate, the sample was dialyzed in the same buffer as for the size exclusion.

### Identification of anti-BCLA circulating antibodies by Western blot

Single Western blot strips were prepared using 15-well 4–12% NuPage gels (Life technologies) loaded with 5 μl of sample at 0.1 mg/mL. The gels were run at 185 V for 40 min in MES buffer then electro-transferred at 105 V for 1.5 h on PVDF membranes. The transferred lanes were then cut out into individualized strips. The strips were then blocked in Tris buffer saline with Tween (TTBS) with 5% powdered milk (w/v) for 1 h. Serum testing was then performed in TTBS with a dilution of 1:400 of the serum for 1 h at 4 °C. The strips were then extended washed in TTBS and further incubated 1 h with a 1:7500 dilution of secondary antibody targeting either mouse IgGs or human IgGs and coupled with a phosphatase alkaline enzyme (Promega). Following extended TTBS wash steps, the blots were revealed by the addition of the chromogenic substrate at room temperature (RT) (Invitrogen). Bands in the positive sera appear within 1 to 5 min. In addition to the serum testing, a single strip was always used as an internal antigen control for each blot set. After blocking, this strip was incubated for 1 h with a peroxidase coupled anti poly-histidine monoclonal antibody (Sigma) diluted 1:2000 in TTBS. After extended wash steps in TTBS, the blot was revealed using the SigmaFast DAB with metal enhancers (Sigma). For each series of i.p. or orally infected mice, a serum of at least one mouse was checked for *Toxoplasma* antibodies using Western blot analysis of the IgG immune response using the commercial kit LD bio *Toxoplasma* mouse IgG (LD bio), with the same anti-mouse IgG-alkaline phosphatase conjugate and chromogenic substrate previously described as for BCLA.

### BCLA ELISA

#### Peptide synthesis

The following BCLA peptides were synthetized by Genscript with N-terminal Acetyl groups:

AB_F: Nter-MERPAAGSMEKEKPVLPGEGEGLPKHETKPALTDEKRTKPGGP-Cter.

A3_B: Nter-AAGSMEKDKLVLPGE-Cter.

#### Plate preparation

Midisorp plates (Nunc) were coated overnight (O.N) at 4 °C with rBCLA, peptides AB_F and A3_B all at 2 μg/mL in 100 mM calcium carbonate buffer pH 9.6 with 100 μl per well. After coating, plates were washed twice with 350 μl of DPBS 0.05% Tween 20 (DPBS/Tween) then blocked for at least 2 h with 300 μl Superblock blocking buffer (Thermo Fisher) after which the buffer was removed and the plates dried upside down. Once dried, the plates could be stored for extended periods of time at 4 °C with no loss in serological reactivity.

#### Sample preparation

All serum dilutions were prepared in DPBS 0.05% Tween 20, 0.1% BSA no more than 2 h prior to the assay. For both mouse and human tested sera, 1:400 dilutions were prepared. Eleven standards were also freshly prepared in both tests, consisting of 10 serial dilutions of a positive frozen stock serum set at 100 UI. Starting at a dilution 1:200 and following a ¾ dilution increment, the following titration points were prepared: 200 UI (1:200), 150 UI (1:266), 112.5 UI (1:356), 84.4 UI (1:474), 63.3 UI (1:632), 47.5 UI, (1:843) 35.6 UI (1:1124), 26.7 UI (1:1498), 20 UI (1:1998), and 15 UI (1:2663). A 0 UI standard was prepared with a seronegative serum diluted at 1:400.

#### Assay

All the subsequent steps were implemented on the Gemini ELISA automation platform (Stratec) but could also be performed manually at RT. Dried plates were first washed twice with 350 μl of DPBS/Tween. Dilutions of the tested sera and standards were then distributed in the plates as row duplicates with 100 μl per well. Plates were then incubated 1 h at RT. After the incubation period, plates were washed 4 times with 350 μl of DPBS/Tween, 100 μl of peroxidase coupled secondary antibody dilution (1:50,000 anti-mouse IgG or 1:60,000 anti-human IgG, Sigma Aldrich ref A0168 and A0170 respectively) in DPBS 0.05% Tween 20, 0.1% BSA were then rapidly distributed in all wells. After 1 h at RT, plates were washed 4 times in DPBS-Tween. Revelation reaction were performed by adding 100 μl of TMB Substrate (Thermo Fisher ref 34029) for 20 min precisely at RT then stopping the reaction with 50 μl of H_2_SO_4_ 0.2 M followed by 30 s of mixing. Well absorbance measurement was then performed using the Gemini integrated spectrophotometer at 450 nm.

#### Data treatment

Blank subtractions were performed on duplicate blank wells for which where primary antibody/sera were omitted but treated similarly as the others for all subsequent steps (washes, secondary Ab, substrate). Standard serum dilutions were averaged and fitted with a 4-parameter logistic regression with the upper asymptote value (*D*_*i*_) fixed at 2.5 AU and all other variables (*A*_*i*_, *B*_*i*_, *C*_*i*_) allowed to fit. From this regression, tested dilution duplicates could have their apparent UI calculated and averaged; if in a duplicate measurement the coefficient of variation was observed above 10%, then the sample would be re-tested. All the ELISA data presented in this work was obtained several times in independent titrations.

### Peptide dot blot screening

Dot blot peptide assays were custom synthetized by JPT Peptide technologies on cellulose membranes with *N*-acetyl moieties on the N-terminus. Two sets of membranes were screened: (1) covering the rBCLA region (res 1089-1275) with a total of 59 peptides, each 15 aa long with an overlap of 12 and an offset of 3; (2) covering repeat 4 (res 446-493) with a total of 18 peptides, each 15 aa long with an overlap of 12 and an offset of 3. Dot blot assays were performed as described by the manufacturer. Briefly, the membranes were first activated 5 min in 100% ethanol then washed 3 times 3 min in DPBS-Tween. Blocked O. N at 4 °C in DPBS-Tween 0.5% powdered milk then washed again 3 times 3 min in DPBS-Tween. Tested sera were diluted to 1/400 in DPBS-Tween 0.1% BSA and incubated for 3 h at RT with the membrane. Following a 3 times 3 min DPBS-Tween wash, the membranes were incubated with anti-IgG peroxidase coupled Ab (Sigma A0170) diluted to 1/100,000 for 2 h at RT. Following a 3 times 3 min wash in DPBS-Tween, the membrane was briefly immerged in the freshly prepared SuperSignal West Pico Chemiluminescent Substrate (Thermo Fisher) and revealed using the C-Digit (Licor) scanner. Dot intensity was integrated using ImageJ. For data analysis of independent dot blots, integrated intensities from every peptide dot [*I*_(*p*)_] were normalized into enrichment factors Fe_(*p*)_ using the baseline integrated intensity of peptide 59 [*I*_(*p* = 59)_] which never reacts with any sera. The following can be expressed with the following equation:

$$ {\mathrm{Fe}}_{(p)}=\raisebox{1ex}{${I}_{(p)}$}\!\left/ \!\raisebox{-1ex}{${I}_{\left(p=59\right)}$}\right. $$

where *p* represents the peptide number.

In order to increase the reactivity score over 5 independent serum blots (from patients positive to rBCLA by Western blot) symbolized as (+), Fe_(*p*)_ enrichment scores were summed with each other, and to subtract the non-specific reactivity, the same sum was performed on 5 negative serum (all from Sag1-negative patients) peptides, symbolized as (−) and subtracted. Peptide reactivity scores can be expressed though the following equation:

$$ {\mathrm{Rs}}_{(p)}=\left[\sum {\left({\mathrm{Fe}}_{(p)}\right)}^{\left(+\right)}-\sum {\left({\mathrm{Fe}}_{(p)}\right)}^{\left(-\right)}\right] $$

where Rs_(*p*)_ is the total reactivity score at a specific peptide position.

### Human sera

Human sera were retrospectively selected from the biobank collection of the Parasitology-Mycology Clinical Laboratory in Grenoble Alpes University Hospital in France. This biobank is registered with the French Ministry of Health under the number DC-2008-582. The Quality Management System is applied in the Parasitology-Mycology Clinical Laboratory in the University Grenoble Alpes Hospital and our laboratory is accredited according to ISO 15189 standard. All the sera were collected in agreement with the institution and after no patient’s opposition. The selected sera were stored for toxoplasmosis serological routine analysis between January 1, 2014, and May 1, 2018. The analyses with Vidas® Toxo IgM and IgG (bioMérieux, France) and Architect Toxo IgG and IgM (Abbott, Germany) were performed in the Parasitology-Mycology Clinical Laboratory of Grenoble Alpes University Hospital. Briefly, the antibody titers for IgG were quantitatively expressed in IU/mL whereas IgM were expressed as an index. The cutoffs defined by Vidas®, bioMérieux manufacturer were as follows: (i) IgG (IU/mL): negative < 4.0; 4.0 ≤ equivocal (gray zone) < 8; ≥ 8 positive; (ii) IgM (index): negative < 0.55; 0.55 ≤ equivocal (gray zone) < 0.65; ≥ 0.65 positive. The cutoffs defined by Architect®, Abbott manufacturer were as follows: (i) IgG (IU/mL): negative < 1.6; 1.6 ≤ equivocal (gray zone) < 3.0; ≥ 3.0 positive; (ii) IgM (index): negative < 0.50; 0.50 ≤ equivocal (gray zone) < 0.60; ≥ 0.60 positive [29]. BCLA titers were measured on 156 selected patients’ sera corresponding to five different biological and clinical forms of toxoplasmosis (Additional file 7: Table S2 and Table 1): non-immunized patients against toxoplasmosis (seronegative, *n* = 43), patients with past immunity (chronic toxoplasmosis, *n* = 79), patients with proved ocular toxoplasmosis (ocular toxoplasmosis, *n* = 18), and immunocompromised patients with asymptomatic serological reactivation (serological reactivation, *n* = 5) or with toxoplasmosis disease (disseminated toxoplasmosis, *n* = 7). The absence of *T. gondii* immunity was concluded when the IgM- and IgG-specific antibody levels measured using Architect Toxo® IgG and Toxo® IgM assays were negative. Past immunity was considered when IgG were above the threshold of positivity with at least one method, and the IgM were negative or weakly positive (Architect® and Vidas®). Proved ocular toxoplasmosis (OT) was confirmed by detection of either Toxoplasma DNA using PCR or a local production of IgG and/or IgA antibodies by Western blot (LDBio®) [37]. Cerebral (*n* = 5), pulmonary (*n* = 2), disseminated (*n* = 1), and acute toxoplasmosis (*n* = 1) were diagnosed by PCR in cerebrospinal fluid, brain biopsy, bronchoalveolar fluid, and/or buffy coat in nine immunocompromised patients (acquired immunodeficiency syndrome, allogeneic hematopoietic stem cell transplant and kidney transplant recipients). These patients had clinical symptoms and radiological evidence of active disease [7, 38]. Asymptomatic serological reactivations were observed in immunocompromised patients during the serological follow-up. In these five patients, an increase of IgG levels compared to previous serological results were observed; furthermore, the *Toxoplasma*-PCR performed were negative, and these patients did not develop any clinical signs of toxoplasmosis [39].

### Protein purification-, immunoblotting-, and mass spectrometry-based proteomic analysis

PruΔ*ku80*-BCLA-HAFlag-infected host HFF cell extracts containing Flag-tagged protein were incubated with anti-FLAG M2 affinity gel (Sigma-Aldrich) for 1 h at 4 °C. Beads were washed with a 10 CV of BC500 buffer (20% glycerol, 20 mM Tris-HCl pH 8.0, 500 mM KCl, 0.05% NP-40, 100 mM PMSF (phenylmethyl sulfonyl fluoride), 0.5 mM DTT, and 1× protease inhibitors). Bound peptides were eluted stepwise with 250 g/mL FLAG peptide (Sigma-Aldrich) diluted in BC100 buffer. Protein bands were excised from colloidal blue-stained gels (Thermo Fisher Scientific), treated with DTT and iodoacetamide to alkylate the cysteines before in-gel digestion using modified trypsin (Sequencing grade; Promega). The resulting peptides from individual bands were analyzed by online nanoLC-MS/MS (UltiMate 3000 coupled to LTQ-Orbitrap Velos Pro; Thermo Fisher Scientific) using a 25-min gradient. Peptides and proteins were identified and quantified using MaxQuant (version 1.5.3.17) through concomitant searches against ToxoDB (20151112 version), and the frequently observed contaminant database embedded in MaxQuant. The minimum peptide length was set to 7 amino acids. The minimum number of peptides, razor + unique peptides, and unique peptides was all set to 1. Maximum false discovery rates were set to 0.01 at peptide and protein levels.

### Mass spectrometry-based proteome-wide analyses

HFF cells were grown to confluence and infected with type I (RHΔ*ku80* or RHΔ*ku80* MORC KD) or type II (PruΔ*ku80* or PruΔ*ku80* MORC KD) strains before lysis in 8 M urea and 50 mM HEPES. Extracted proteins were reduced using 20 mM of dithiothreitol for 1 h at 37 °C before alkylation with 55 mM of iodoacetamide for 45 min at room temperature in the dark. The samples were then diluted using ammonium bicarbonate to obtain a urea concentration of 4 M. Proteins were digested with LysC (Promega) at a ratio of 1:200 during 4 h at 37 °C. The samples were diluted again using ammonium bicarbonate to obtain a urea concentration of 1 M. Proteins were then digested with Trypsin (Promega) at a ratio of 1:200 overnight at 37 °C. The resulting peptides were purified by C18 reverse phase chromatography (Sep-Pak C18, Waters) before drying down before fractionation by tip-based strong cation exchange (3 M Empore). For this, peptides were dissolved in 5% acetonitrile, 1% TFA and eluted in 4 fractions (F1: 100 mM ammonium acetate, 20% ACN, 0.5% formic acid; F2: 175 mM ammonium acetate, 20% ACN, 0.5% formic acid; F3: 375 mM ammonium acetate, 20% ACN, 0.5% formic acid; F4: 80% acetonitrile, 5% ammonium hydroxide) before desalting using C18 reverse phase chromatography (Ultra-Micro SpinColumns, Harvard Apparatus). Technical triplicates were performed. NanoLC-MS/MS analyses were performed using an Ultimate 3000 RSLCnano coupled to a Q-Exactive Plus (Thermo Scientific). Peptides were sampled on a 300 μm × 5 mm PepMap C18 precolumn and separated on a 75 μm × 250 mm PepMap C18 column (Thermo Scientific) using a 120-min gradient. MS and MS/MS data were acquired using the Xcalibur software (Thermo Scientific). RAW files were processed using MaxQuant^48^ version 1.6.2.10. Spectra were searched against the *Toxoplasma gondii* database (ME49 taxonomy, version 30 downloaded from ToxoDB, the Uniprot database (*Homo sapiens* taxonomy, April 2019 version), the frequently observed contaminants database embedded in MaxQuant, and the corresponding reverse databases. Trypsin was chosen as the enzyme and two missed cleavages were allowed. Precursor and fragment mass error tolerances were set at their default values. Peptide modifications allowed during the search were carbamidomethyl (C, fixed), acetyl (protein N-term, variable), and oxidation (M, variable). The minimum number of peptides and razor + unique peptides was set to 1. Maximum false discovery rates were set to 0.01 at PSM and protein levels. The match between runs option was activated. Statistical analyses were performed using ProStaR. Peptides and proteins identified in the reverse, and contaminant databases or matching to human sequences were discarded. Only proteins quantified in at least 3 replicates of one condition were conserved. After log2 transformation, protein intensities were normalized using the summed intensities of a pool of proteins for which transcripts were found invariable in RNA-seq dataset (0.91 < fold change < 1.1). Missing values were then imputed (slsa method for Partially Observed Values and DetQuantile with quantile set to 2.5 and factor set to 1 for missing entirely in the condition). Statistical testing was conducted using limma. Differentially abundant proteins were sorted out using the following cutoffs: log2(fold change) ≥ 0.8 or ≤ − 0.8 and *P* values allowing to reach an FDR ~ 1% according to the Benjamini-Hochberg estimator. The mass spectrometry proteomics data have been deposited to the ProteomeXchange Consortium via the PRIDE partner repository with the dataset identifier PXD016845.

### Chromatin Immunoprecipitation and next-generation sequencing in *T. gondii*

HFF cells were grown to confluence and infected with type II (Pru*∆ku80* or Pru*∆ku80* MORC KD) strains. Harvested intracellular parasites were crosslinked with formaldehyde (final concentration 1%) for 8 min at room temperature, and the crosslinking was stopped by addition of glycine (final concentration 0.125 M) for 5 min at room temperature. Crosslinked chromatin was lysed in ice-cold lysis buffer (50 mM HEPES KOH pH 7.5, 14 0 mM NaCl, 1 mM EDTA, 10% glycerol, 0.5% NP-40, 0.125% Triton X-100, protease inhibitor cocktail) and sheared in shearing buffer (1 mM EDTA pH 8.0, 0.5 mM EGTA pH 8.0, 10 mM Tris pH 8.0, protease inhibitor cocktail) by sonication using a Diagenode Biorupter. Samples were sonicated by 16 cycles of 30 s ON and 30 s OFF, to 200–500 base-pair average size. Immunoprecipitation was carried out using sheared chromatin, 5% BSA, protease inhibitor cocktail, 10% Triton X-100, 10% deoxycholate, DiaMag Protein A-coated magnetic beads (Diagenode), and antibodies targeting histone PTM or protein of interest. A rabbit IgG antiserum was used as a control mock. After overnight incubation at 4 °C on a rotating wheel, chromatin-antibody complexes were washed and eluted from beads by using iDeal ChIP-seq kit for Histones (Diagenode) according to the manufacturer’s protocol. Samples were de-crosslinked by heating for 4 h at 65 °C. DNA was purified by using the IPure kit (Diagenode) and quantified by using Qubit Assays (Thermo Fisher Scientific) according to the manufacturer’s protocol. For ChIP-seq, purified DNA was used to prepare libraries and then sequenced by Arraystar (USA).

### Library preparation, sequencing, and data analysis (Arraystar)

ChIP-Sequencing library preparation was performed according to Illumina’s protocol Preparing samples for ChIP sequencing of DNA. **Library preparation:** 10 ng DNA of each sample was converted to phosphorylated blunt-ended with T4 DNA polymerase, Klenow polymerase, and T4 polymerase (NEB); An ‘A’ base was added to the 3′ end of the blunt phosphorylated DNA fragments using the polymerase activity of Klenow (exo minus) polymerase (NEB); Illumina’s genomic adapters were ligated to the A tailed DNA fragments; PCR amplification was performed to enrich ligated fragments using Phusion High Fidelity PCR Master Mix with HF Buffer (Finnzymes Oy). The enriched product of ~ 200–700 bp was cut out from the gel and purified. **Sequencing:** The library was denatured with 0.1 M NaOH to generate single-stranded DNA molecules and loaded onto channels of the flow cell at 8 pM concentration, amplified in situ using TruSeq Rapid SR cluster kit (#GD-402-4001, Illumina). Sequencing was carried out by running 100 cycles on Illumina HiSeq 4000 according to the manufacturer’s instructions. **Data analysis:** after the sequencing platform generated the sequencing images, the stages of image analysis and base calling were performed using the Off-Line Basecaller software (OLB V1.8). After passing the Solexa CHASTITY quality filter, the clean reads were aligned to *T. gondii* reference genome (Tgo) using BOWTIE (V2.1.0). Aligned reads were used for peak calling of the ChIP regions using MACS V1.4.0. Statistically significant ChIP-enriched regions (peaks) were identified by comparison of two samples, using a *P* value threshold of 10^−5^. Then, the peaks in each sample were annotated by the overlapped gene using the newest *T. gondii* database. The EXCEL/BED format file containing the ChIP-enriched regions was generated for each sample. **Data visualization:** the mapped 100 bp reads represent enriched DNA fragments by the ChIP experiment. Any region of interest in the raw ChIP-seq signal profile can be directly visualized in a genome browser. We use 10-bp resolution intervals (10-bp bins) to partition the stacked reads region and count the number of reads in each bin. All the 10-bp resolution ChIP-seq profiles of each sample are saved as UCSC wig format files, which can be visualized in *T. gondii* Genome Browser (http://protists.ensembl.org/Toxoplasma_gondii/Info/Index). All these raw and processed files can be found at Series GSE136060.

### RNA-seq and sequence alignment

Total RNAs were extracted and purified using TRIzol (Invitrogen, Carlsbad, CA, USA) and RNeasy Plus Mini Kit (Qiagen). RNA quantity and quality were measured by NanoDrop 2000 (Thermo Scientific). RNA integrity was assessed by standard non-denaturing 1.2% TBE agarose gel electrophoresis. The ribosomal large subunits from *Toxoplasma* and the host cells were used to verify that the ratio between *Toxoplasma* RNA versus host RNA was equivalent between the different biological samples, thus indicating that the samples had equivalent infection rates. For each condition, total RNAs from two independent biological replicates were pooled to prepare cDNA libraries which were then sequenced using Illumina technology in a single replicate dataset. RNA sequencing was performed by GENEWIZ (South Plainfield, NJ, USA). Briefly, the RNA quality was checked with an Agilent 2100 Bioanalyzer (Agilent Technologies, Palo Alto, CA, USA) and Illumina TruSEQ RNA library prep and sequencing reagents were used following the manufacturer’s recommendations (Illumina, San Diego, CA, USA). The samples were paired-end multiplex sequenced (2 × 125 bp) on the Illumina Hiseq 2500 platform and generated at least 70 million reads for each sample. The RNA-seq reads (FASTQ) were processed and analyzed using the Lasergene Genomics Suite version 14 (DNASTAR, Madison, WI, USA) using default parameters. The paired-end reads were uploaded onto the SeqMan NGen (version 14, DNASTAR. Madison, WI, USA) platform for reference-based assembly using either the *Mus musculus* genome package (GRCm38.p3) or the *Toxoplasma* type II ME49 strain (ToxoDB-24, ME49 genome) as reference template. The ArrayStar module (version 14, DNASTAR. Madison, WI, USA) was used for normalization, differential gene expression and statistical analysis of uniquely mapped paired-end reads using the default parameters. The expression data quantification and normalization were calculated using the RPKM (reads per kilobase of transcript per million mapped reads) normalization method. All these raw and processed files can be found at Series GSE136123.

## Supplementary Information

**Additional file 1: Figure S1.** Sequence alignment of BCLA proteins. The protein sequences of BCLA from *Toxoplasma gondii* RH, ME49 and VEG strains along with the partial sequence from *Hammondia hammondi* (HHA_209755 and HHA_462480) were aligned. Matching and different residues are shown as dots and in blue, respectively. The red box represents the signal peptide, while the yellow and green boxes represent the central repeats and the blue box the conserved and structured C-terminal domain, referred in the text as rBCLA.

**Additional file 2: Table S1.** *T. gondii* strains, vectors and primers. List of *T. gondii* parasite lines as well as plasmids used in this work. Primers and DNA synthesis construct used in this work are also charted in the table.

**Additional file 3: Figure S2.** rBCLA purification steps. **(a)** Nickel-nitrilotriacetic (Ni-NTA) elution. SDS PAGE electrophoresis of flow though (Ft) and elution fractions 3 to 12. Cter-BCLA (rBCLA) is shown by the black arrow migrating above the 17 kDa molecular weight marker and enriched during Ni-NTA elution. **(b)** Mono-Q anion exchange chromatography. Gradient elution chromatogram with the 280 nm absorbance curve in blue and conductivity curve in orange. The elution fraction numbers are numbered on the chromatogram in light grey. The SDS PAGE gel colored by Coomassie Blue is shown with the sample (load) followed by the elution fractions F31 to F49. rBCLA is shown by a black arrow. Fractions kept for further purification are highlighted using a dotted bar. **(c)** Size exclusion chromatography. Chromatogram following 280 nm absorbance (blue curve) as a function of elution volume with sampled fractions in light grey. Superposed is the SDS PAGE electrophoresis showing the previous step pool prior to concentration (Load) and eluted fractions. rBCLA is shown by a black arrow. Fractions kept for further purification are highlighted using a dotted bar.

**Additional file 4: Figure S3.** Western blot evidence that rBCLA is reacting with sera of mice infected by cystogenic strains. Single western-blot strips were loaded with 0.5 μg of recombinant rBCLA. The strips were tested on sera collected from mice in acute and chronic phase of toxoplasmosis with various *T. gondii* strains, route of infection and mice genetic background. **(a)** Sera were collected from C57BL/6 mice 8 days post orally inoculation of 47 cysts of either type II (76K-GFP-luc)-WT or Δ*bcla* strains. **(b)** Sera were collected from BALB/c mice 42 days post-intraperitoneal inoculation of 10^5^ tachyzoites of the type II (76K-GFP-luc) strain. Reactivation was induced in chronically infected mice using corticosteroids. The serological status of the infected mice was determined by immunoblot using the LDBio Diagnostics test (lower panels).

**Additional file 5: Figure S4.** BCLA reactivity in human sera. **(a)** rBCLA reactivity detected by western blot. Serological reactivity is assessed by qualifying the presence or absence of the rBCLA band, positively revealed in black by anti-histidine HRP-conjugated Ab (his-rBCLA blot). Positively qualified blots are numbered in blue while negative or ambiguous blots are numbered in black, sample type is detailed by the letter S for serum or AH for aqueous humor. Blots are grouped according to clinical status of the patients. **(b)** Peptidic dot blots for 5 positive sera and one negative serum with peptide numbering and regions covered. On the right, ELISA titrations for rBCLA and SAG1 (Architect®) are shown for these same sera.

**Additional file 6: Figure S5.** Global ELISA reactivity depending on the BCLA antigen composition. Violin plots show the distribution of ELISA reactivities (in UI) of Sag1 negative versus sag1 positive groups. rBCLA titrations are colored in blue while rBCLA combined with peptides ABf and A3b are shown in green. Both titrations were performed on the same sera and are an average of at least two independent measurements. Statistical significance in both cases is measured using a non-parametric Mann-Whitney test. *P* values and their corresponding significance is shown for the comparison between groups.

**Additional file 7: Table S2.** Table of human sera from the biobank. The sera of patients with toxoplasmosis were classified in 5 sheets corresponding to the following pathologies: 1- Toxo immunocompromised, 2- Asymptomatic serological reactivation, 3- Ocular toxoplasmosis, 4- Chronic toxoplasmosis past immunity and 5- seronegative. The serological status is indicated for each serum in an anonymized manner.

## Data Availability

Correspondence and requests for materials should be addressed to M.A.H. All relevant data are enclosed in the manuscript and/or deposited online as outlined here. The RNA-seq demultiplexed FASTQ files and gene-wise quantifications have been deposited in NCBI’s Gene Expression Omnibus and are accessible through GEO Serie accession number GSE136123 [40]. The ChIPseq data have been deposited to the GEO Datasets under accession number GSE136060 [41]. The mass spectrometry proteomics data have been deposited to the ProteomeXchange Consortium via the PRIDE partner repository with the dataset identifier PXD016845 [42].
