# Supplementary figures and images for: A brain cyst load-associated antigen is a Toxoplasma gondii biomarker for serodetection of persistent parasites and chronic infection
Source: BMC Biol. 2021 Feb 9;19:25. doi: 10.1186/s12915-021-00959-9 (PMC7871634; doi:10.1186/s12915-021-00959-9)

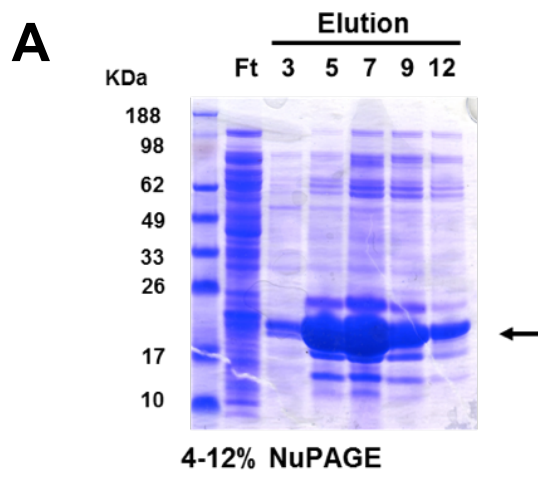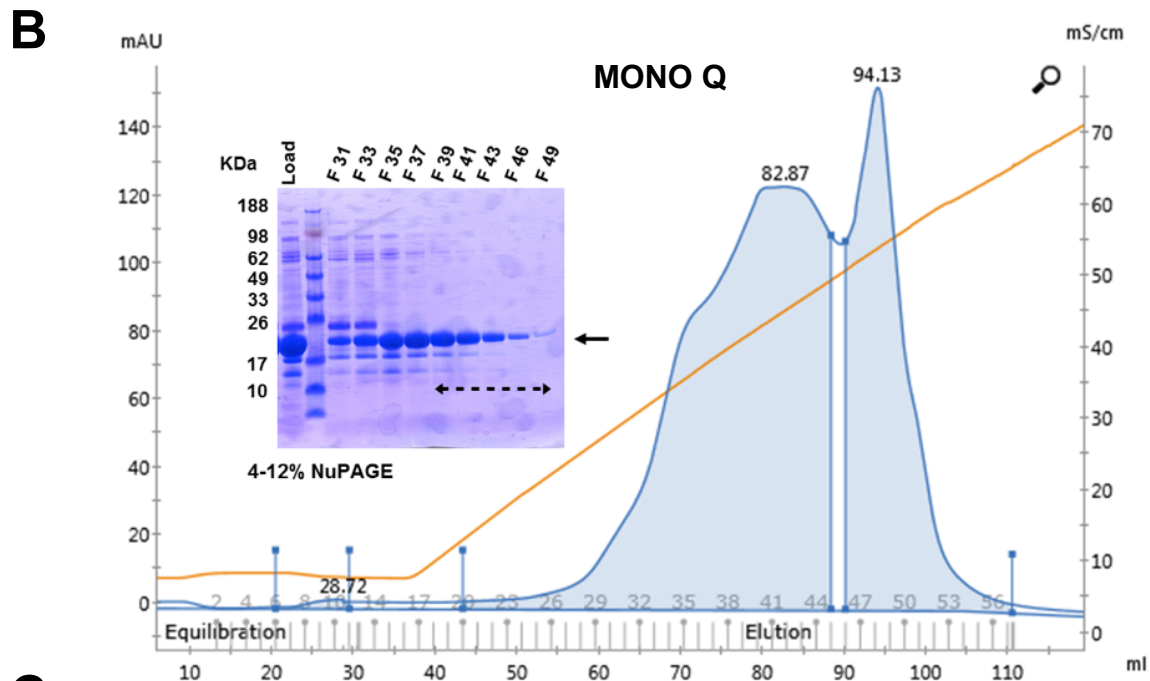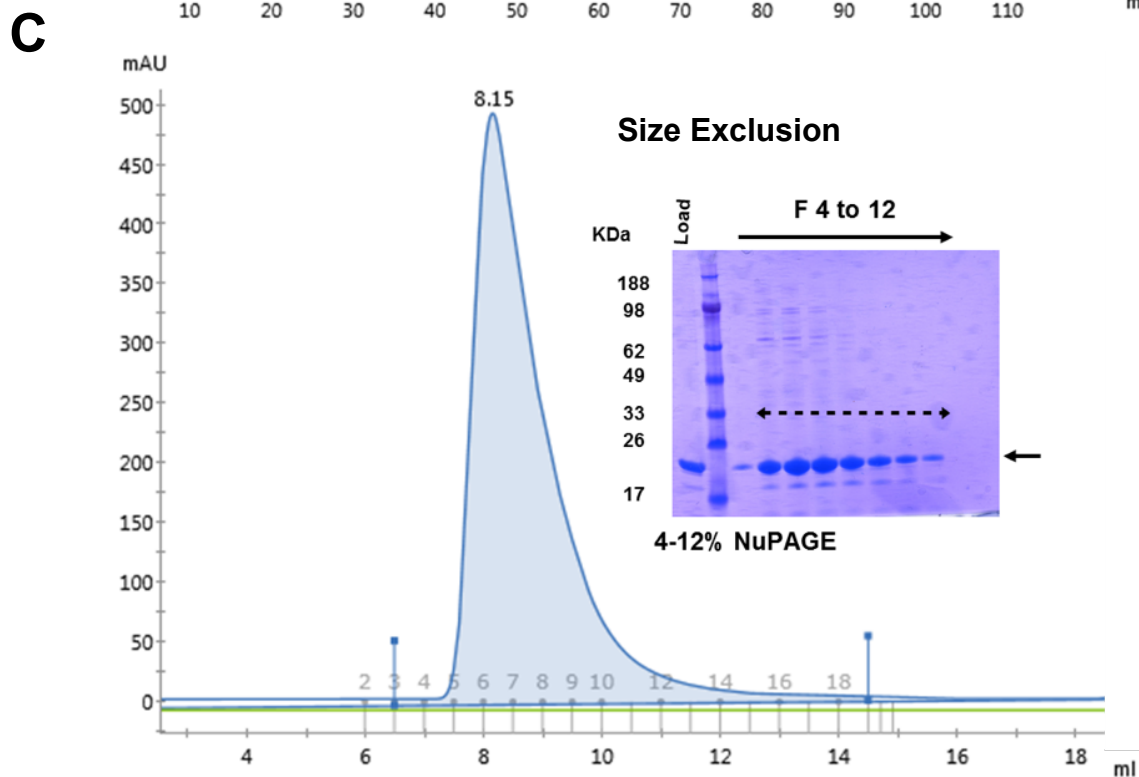

Additional file 3: Figure S2

Supplement: Supplementary file 3 — Additional file 3: Figure S2. rBCLA purification steps. (a) Nickel-nitrilotriacetic (Ni-NTA) elution. SDS PAGE electrophoresis of flow though (Ft) and elution fractions 3 to 12. Cter-BCLA (rBCLA) is shown by the black arrow migrating above the 17 kDa molecular weight marker and enriched during Ni-NTA elution. (b) Mono-Q anion exchange chromatography. Gradient elution chromatogram with the 280 nm absorbance curve in blue and conductivity curve in orange. The elution fraction numbers are numbered on the chromatogram in light grey. The SDS PAGE gel colored by Coomassie Blue is shown with the sample (load) followed by the elution fractions F31 to F49. rBCLA is shown by a black arrow. Fractions kept for further purification are highlighted using a dotted bar. (c) Size exclusion chromatography. Chromatogram following 280 nm absorbance (blue curve) as a function of elution volume with sampled fractions in light grey. Superposed is the SDS PAGE electrophoresis showing the previous step pool prior to concentration (Load) and eluted fractions. rBCLA is shown by a black arrow. Fractions kept for further purification are highlighted using a dotted bar. [file 12915_2021_959_MOESM3_ESM.pdf]

**A**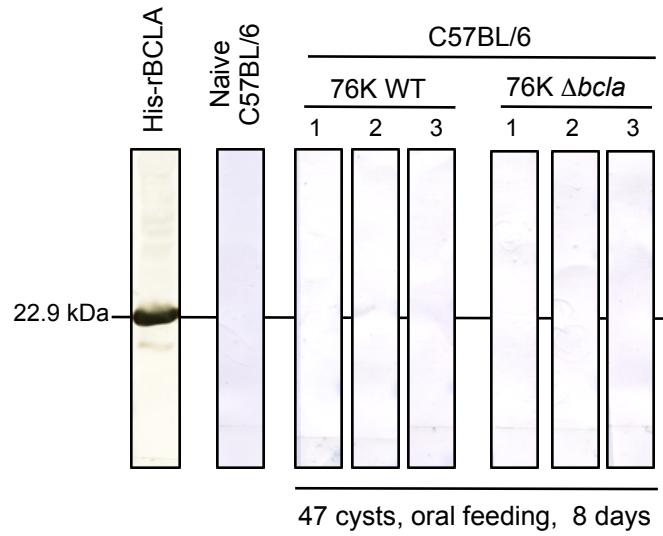**B**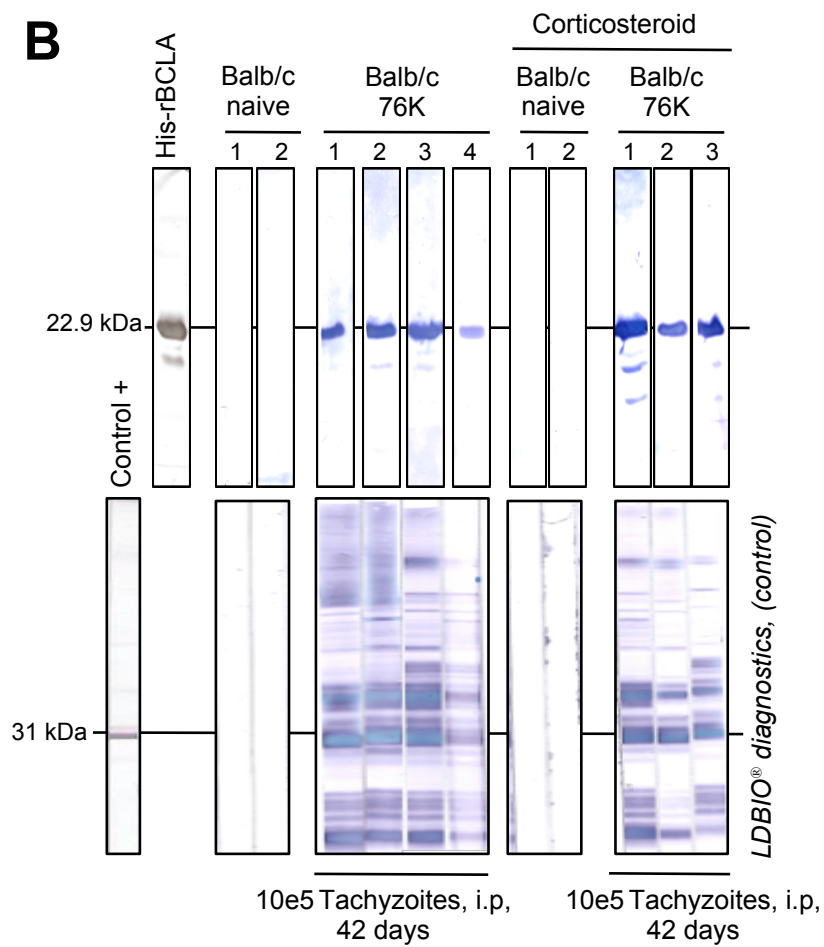

**Additional file 4: Figure S3**

Supplement: Supplementary file 4 — Additional file 4: Figure S3. Western blot evidence that rBCLA is reacting with sera of mice infected by cystogenic strains. Single western-blot strips were loaded with 0.5 μg of recombinant rBCLA. The strips were tested on sera collected from mice in acute and chronic phase of toxoplasmosis with various T. gondii strains, route of infection and mice genetic background. (a) Sera were collected from C57BL/6 mice 8 days post orally inoculation of 47 cysts of either type II (76K-GFP-luc)-WT or Δbcla strains. (b) Sera were collected from BALB/c mice 42 days post-intraperitoneal inoculation of 105 tachyzoites of the type II (76K-GFP-luc) strain. Reactivation was induced in chronically infected mice using corticosteroids. The serological status of the infected mice was determined by immunoblot using the LDBio Diagnostics test (lower panels). [file 12915_2021_959_MOESM4_ESM.pdf]

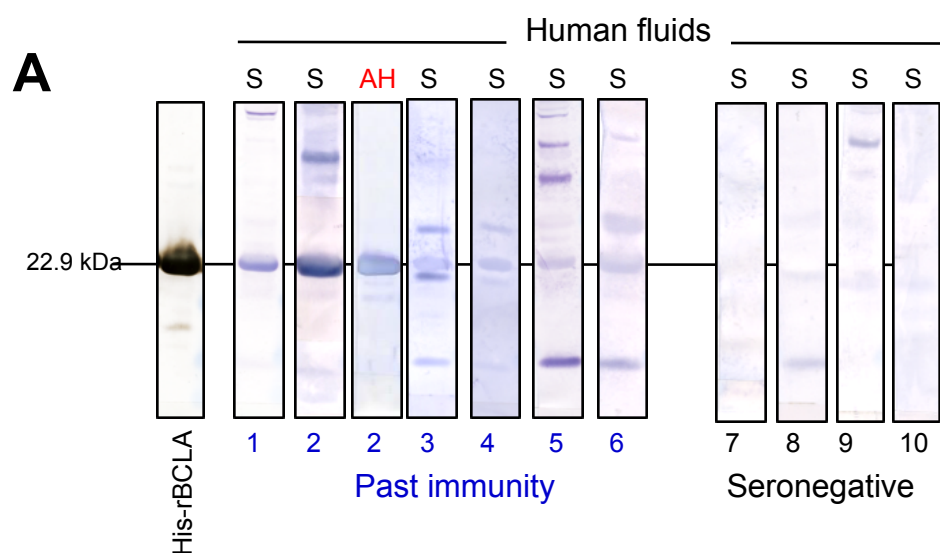

**B**

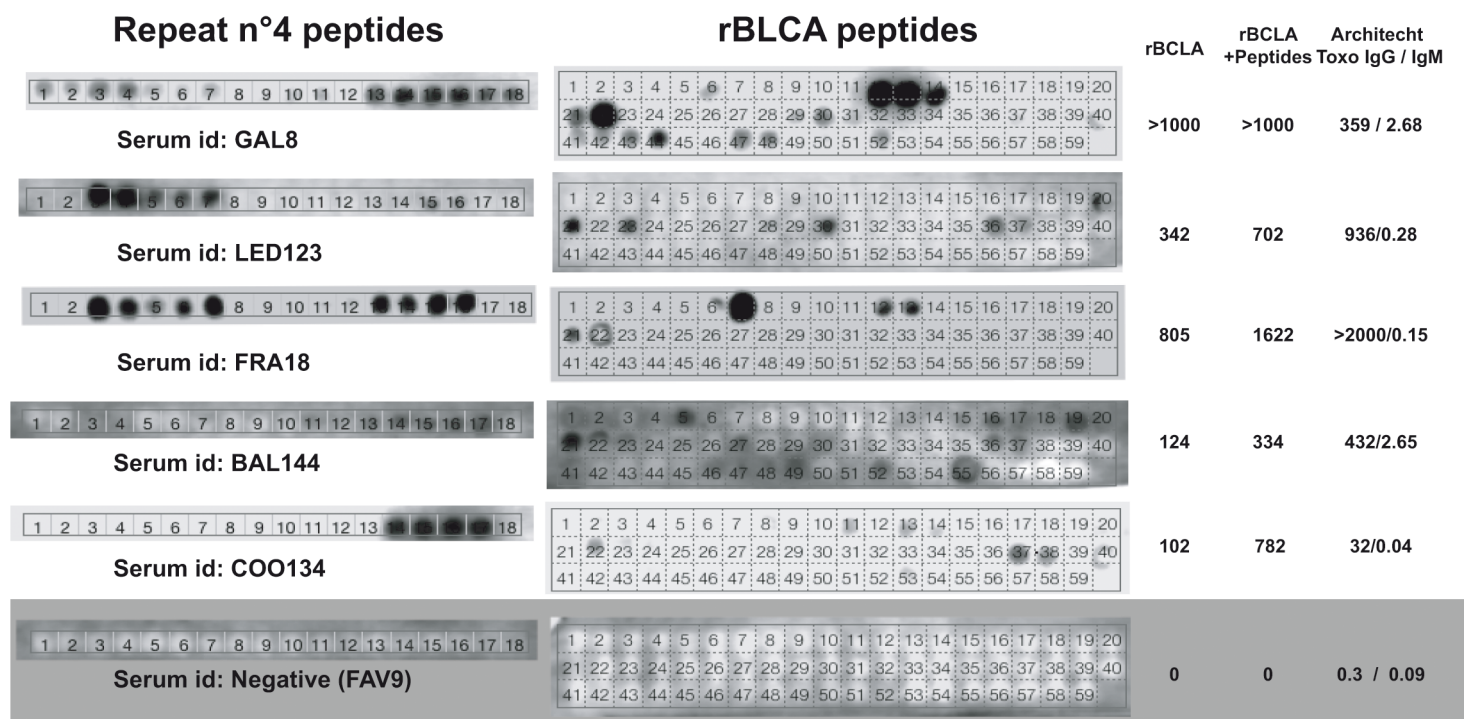

**Additional file 5: Figure S4**

Supplement: Supplementary file 5 — Additional file 5: Figure S4. BCLA reactivity in human sera. (a) rBCLA reactivity detected by western blot. Serological reactivity is assessed by qualifying the presence or absence of the rBCLA band, positively revealed in black by anti-histidine HRP-conjugated Ab (his-rBCLA blot). Positively qualified blots are numbered in blue while negative or ambiguous blots are numbered in black, sample type is detailed by the letter S for serum or AH for aqueous humor. Blots are grouped according to clinical status of the patients. (b) Peptidic dot blots for 5 positive sera and one negative serum with peptide numbering and regions covered. On the right, ELISA titrations for rBCLA and SAG1 (Architect®) are shown for these same sera. [file 12915_2021_959_MOESM5_ESM.pdf]

## rBCLA vs rBCLA/Peptides Elisa reactivity

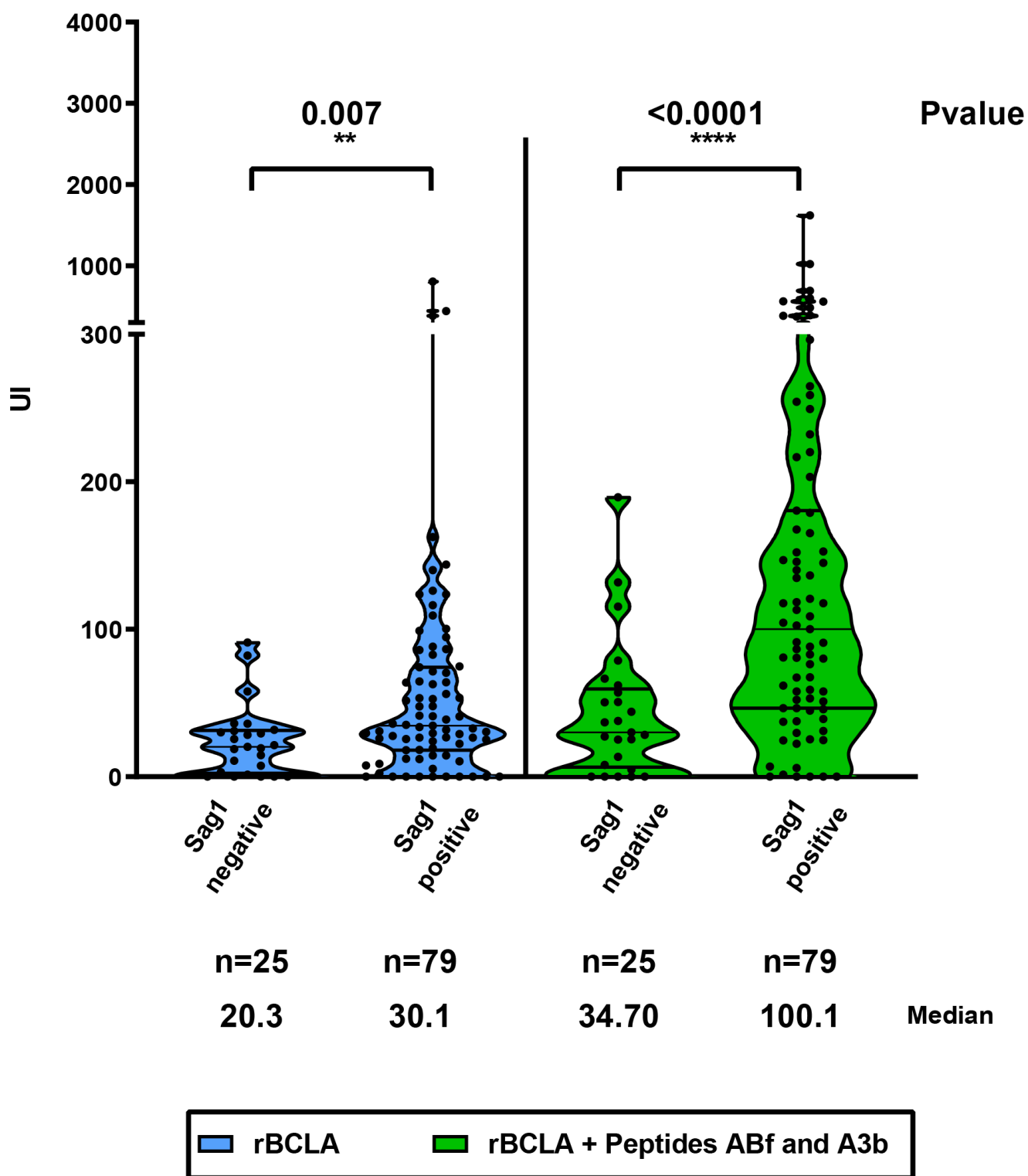

Additional file 6: Figure S5

Supplement: Supplementary file 6 — Additional file 6: Figure S5. Global ELISA reactivity depending on the BCLA antigen composition. Violin plots show the distribution of ELISA reactivities (in UI) of Sag1 negative versus sag1 positive groups. rBCLA titrations are colored in blue while rBCLA combined with peptides ABf and A3b are shown in green. Both titrations were performed on the same sera and are an average of at least two independent measurements. Statistical significance in both cases is measured using a non-parametric Mann-Whitney test. P values and their corresponding significance is shown for the comparison between groups. [file 12915_2021_959_MOESM6_ESM.pdf]
