# Supplementary material for: A brain cyst load-associated antigen is a Toxoplasma gondii biomarker for serodetection of persistent parasites and chronic infection
Source: BMC Biol. 2021 Feb 9;19:25. doi: 10.1186/s12915-021-00959-9 (PMC7871634; doi:10.1186/s12915-021-00959-9)
Supplement: Supplementary file 1 — Additional file 1: Figure S1. Sequence alignment of BCLA proteins. The protein sequences of BCLA from Toxoplasma gondii RH, ME49 and VEG strains along with the partial sequence from Hammondia hammondi (HHA_209755 and HHA_462480) were aligned. Matching and different residues are shown as dots and in blue, respectively. The red box represents the signal peptide, while the yellow and green boxes represent the central repeats and the blue box the conserved and structured C-terminal domain, referred in the text as rBCLA. [file 12915_2021_959_MOESM1_ESM.pdf]

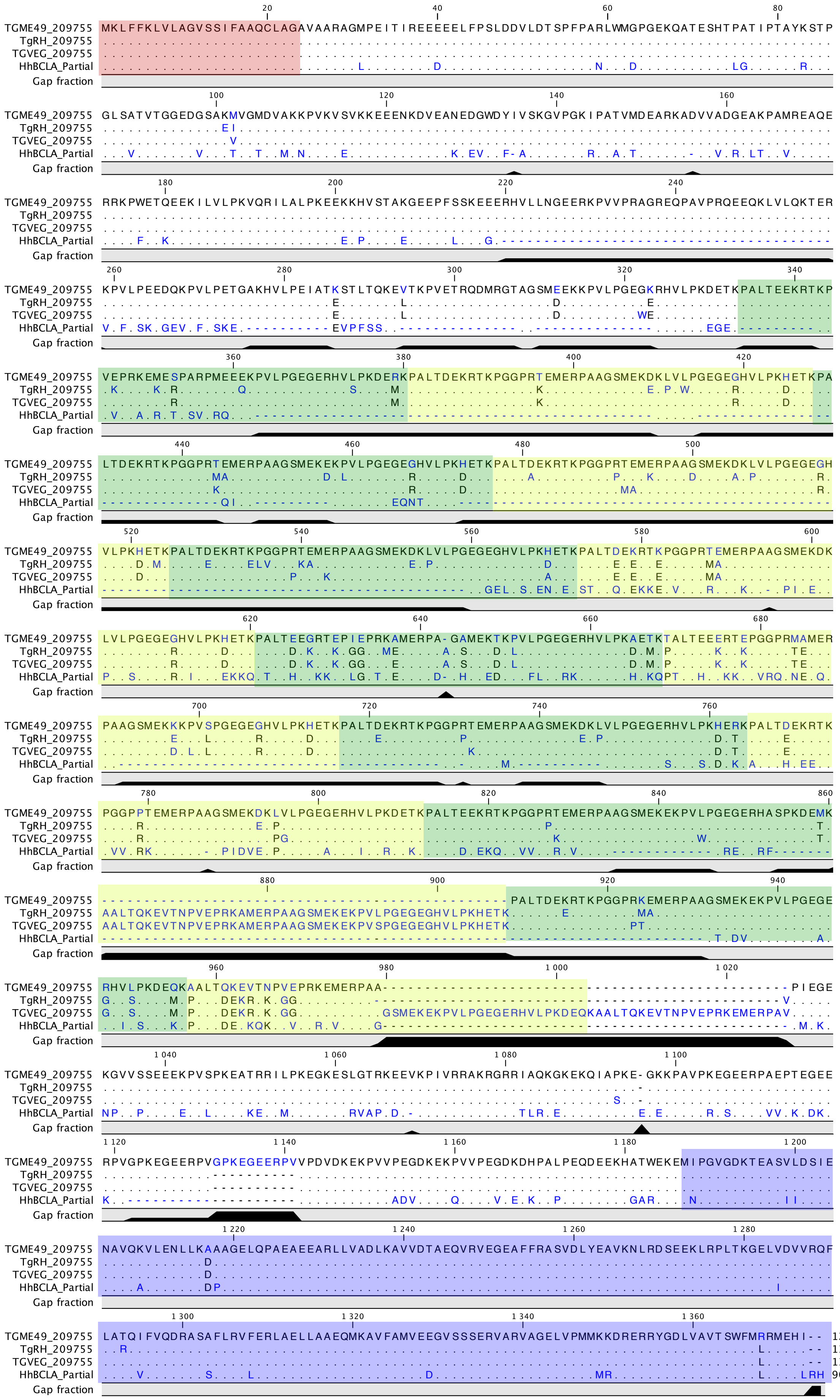

Additional file 1: Figure S1
